# Genome-wide identification, genomic organization, and expression profiling of the *CONSTANS-like (COL)* gene family in petunia under multiple stresses

**DOI:** 10.1186/s12864-021-08019-w

**Published:** 2021-10-08

**Authors:** Khadiza Khatun, Sourav Debnath, Arif Hasan Khan Robin, Antt Htet Wai, Ujjal Kumar Nath, Do-Jin Lee, Chang-Kil Kim, Mi-Young Chung

**Affiliations:** 1 Department of Biotechnology, Patuakhali Science and Technology University, Patuakhali, 8602 Bangladesh; 2 Department of Biochemistry and Food Analysis, Patuakhali Science and Technology University, Patuakhali, 8602 Bangladesh; 3 Department of Genetics and Plant Breeding, Bangladesh Agricultural University, Mymensingh, 2202 Bangladesh; 4 Department of Biology, Yangon University of Education, Kamayut Township, 11041 Yangon, Yangon Region Myanmar; 5 Department of Agricultural Education, Sunchon National University, 255 Jungangno, Suncheon, Jeonnam 57922 Republic of Korea; 6 Department of Horticulture, Kyungpook National University, Daegu, South Korea

**Keywords:** Petunia, Genome-wide analysis, *Constans*-like gene (*COL*), Expression patterns, Abiotic stresses

## Abstract

**Background:**

CONSTANS-like (CO-like, COL) are putative zinc-finger transcription factors known to play vital role in various plant biological processes such as control of flowering time, regulation of plant growth and development and responses to stresses. However, no systematic analysis of *COL* family gene regarding the plant development and stress response has been previously performed in any solanaceous crop. In the present study, a comprehensive genome-wide analysis of *COL* family genes in petunia has been conducted to figure out their roles in development of organs and stress response.

**Results:**

A total of 33 *COL* genes, 15 *PaCOL* genes in *P. axillaris* and 18 *PiCOL* genes in *P. inflata*, were identified in petunia. Subsequently, a genome-wide systematic analysis was performed in 15 *PaCOL* genes. Considering the domain composition and sequence similarity the 15 *PaCOL* and 18 *PiCOL* genes were phylogenetically classified into three groups those are conserved among the flowering plants. Moreover, all of the 15 PaCOL proteins were localized in nucleus. Furthermore, differential expression patterns of *PaCOL* genes were observed at different developmental stages of petunia. Additionally, transcript expression of 15 *PaCOL* genes under various abiotic and phytohormone treatments showed their response against stresses. Moreover, several *cis-*elements related to stress, light-responsive, hormone signaling were also detected in different *PaCOL* genes.

**Conclusion:**

The phylogenetic clustering, organ specific expression pattern and stress responsive expression profile of conserved petunia *COL* genes indicating their involvement in plant growth and development and stress response mechanism. This work provide a significant foundation for understanding the biological roles of petunia *COL* genes in plant growth, development and in stress response.

**Supplementary Information:**

The online version contains supplementary material available at 10.1186/s12864-021-08019-w.

## Background

Flowering in plants is one of the most important agronomic traits for crop yield and is an indicator of the reproductive success of plants [1]. The control of flowering time is crucial for the completion of pollination, seed development, and as well as adaptation to diverse environmental conditions [2]. CONSTANS (CO) is the central integrator of the photoperiodic flowering control pathways of plants [3]. CO, a nuclear zinc finger transcription factor belonging to the BBX (B-box protein) protein family containing two N-terminus B-Boxes zinc finger domain, and a C-terminal CCT (CO, CO-like and TOC1 (Timing of CAB Expression 1) domain [4]. Different numbers of the CO/CO-like (COL) gene family members were observed among diverse plant species. For example, 17 members were observed in *Arabidopsis* [5], 16 in rice [6], three in wheat [7], 11 in *Chrysanthemum lavandulifolium* [8], 26 in soybean [3], 25 in Chinese cabbage [9]. In plants, COL protein is phylogenetically divided into three groups. Group 1 has two N-terminus B-box domains, one C-terminus CCT domain, and an additional VP motif (valine-proline motif). Group II contains only one N-terminus B-box and a C-terminus CCT domain. Group III contains one full B-box, a diverged zinc finger, and a CCT domain [6, 10, 11].

In *Arabidopsis, CO* was the first cloned CCT gene that regulates the flowering time by regulating the photoperiod pathway [2]. CO mRNA was characterized as a long day (LD)-specific late-flowering mutant phenotype and is regulated by the circadian clock component GI (GIGANTEA) [12]. The CO protein acts in the vascular tissue of leaves and that is involved in determining the timing of xylem expansion during development and in elevation of stomatal opening [13, 14]. CO is a central integrator of both internal circadian clock and the external day-night cycles [3]. Under long-day (LD) conditions the CO proteins stimulate the expression of FT (Flowering Locus T) and SOC1 (Suppressor of Overexpression of CONSTANS 1). The stimulation of FT by CO for the activation of LD- specificity is related to the abundance of CO protein at both transcriptional and post-translational level of regulation [15]. In contrast, the CO homolog Heading date 1 (Hd1) promotes FT expression under SD (short-day) condition but overwhelms the expression of Heading date 3 (HD3) under LD condition [16, 17]. In *Arabidopsis*, CO is transcribed and induced the transcription of FT and TSF (Twin Sister of FT) transcript only in the late afternoon under long-day conditions [18, 19]. It has been reported that the flowering time governed by the CO/FT module is highly conserved among photoperiod-sensitive plants but inconsistent in different species [20–22].

The function of CO may be widely conserved across the angiosperms and not all of these family genes regulate flowering transition or are photoperiod related only rather they have another activity. The biological and developmental functions of CO are observed throughout the life cycle of the plant from the early stages of embryonic development to cotyledons, leaves, shoot apices and inflorescence as well as in developing seeds and shade avoidance response (SAR) [23–26]. In Arabidopsis, AtCOL3 positively regulates the photo-morphogenesis and is involved in root development and anthocyanin accumulation [27], AtCOL5 acts as accelerator of flowering [28]. AtCOL7 regulates branching of the shoot [29]. AtCOL8 expression is observed in the seeds, leaves, flowers, and siliques, AtCOL9 is involved in the flowering time regulation through reducing CO and FT expression [30]. Besides, important functions of *CO* or *COL* genes can also be observed in potato tuber formation, seasonal growth cessation in aspen trees, fruit ripening and stress responses in banana [21, 31, 32]. For example, AtCOL4 acts as a positive regulator for plant tolerance to abiotic stresses and its expression was induced by salt, osmotic and ABA stress [33].

Petunia is one of the most important ornamental plant species and a good model for conducting genetic research [34, 35]. Recently, whole-genome sequencing of petunia species completed that opened a good opportunity of studying the genome-wide identification and analysis of important gene/protein families [36]. Usually most of the petunias are facultative LD plant for flowering although Petunia ‘Wave Purple’ is an obligate LD plant [37–39]. By changing the day length, the flowering time can be controlled to meet up the ever increasing demand of merchandisable flowers throughout the year. The discovery of flowering time and photoperiod responsive gene families could reveal the underlying molecular mechanisms of transferring the day length signal into the flowering time signal. The *COL* gene family has been reported to contribute important function in plant development and stress response other than controlling flowering time. However, any study regarding the evolutionary history, systematic analysis and characterization of the *CO/COL* genes of petunia have never been reported before. A comprehensive analysis of this gene family may enable us to understand the important function of *COL* genes in growth and development and stress response in petunia and establish a foundation for the further study.

## Methods

### Identification of *COL* family genes in petunia species

Protein sequences of COL of *Arabidopsis* and rice were downloaded from TAIR (http://www.arabidopsis.org/) and TIGR (http://rice.plantbiology.msu.edu/) databases respectively. Two approaches were utilized for the identification of COL family proteins in petunia genomes. The whole protein sequences of both *Petunia axillaris* and *Petunia inflata* were retrieved from Sol Genomics Network (SGN, https://solgenomics.net/). The previously identified *Arabidopsis* COL proteins were used as a query to search COL proteins in *Petunia axillaris* and *Petunia inflata* by local blast search tool (https://blast.ncbi.nlm.nih.gov/Blast.cgi) using the Blastp method. The Blast search continued until no more new petunia COL homologs were appeared. A total of 17 and 18 COL homologs were obtained from *Petunia axillaris* and *Petunia inflata* respectively. All candidate sequences were subjected to InterProScan (https://www.ebi.ac.uk/interpro/search/sequence/) and SMART (http://smart.embl-heidelberg.de/) tools with default parameters for annotating of the domain structure. Fifteen out of 17 *Petunia axillaris* and 18 out of 18 *Petunia inflata* COL encoded both B-Box and CCT domains whereas two other proteins of *P. axillaris* encoded only the CCT domain. Secondly, the 15 and 18 genes were verified using HMM (Hidden Markov Model) profile of the CCT domain (PF06203.13) and zinc finger B-box domain (PF00643.23). Finally, all retrieved proteins were submitted to Phytozome database (https://phytozome.jgi.doe.gov/pz/portal.html) to verify and confirm the identified petunia proteins. ORF finder tool (https://www.ncbi.nlm.nih.gov/orffinder/) was utilized to identify the open reading frame (ORF) of petunia *COL* genes. The protein length, molecular weight and isoelectric point of each COL protein were identified using the Expasy-Protopram (https://web.expasy.org/protparam/) web tool. Gene Structure Display Server (GSDS) (http://gsds.gao-lab.org/) was employed to determine the exon–intron distribution of petunia *COL* genes. The Genedoc (www.nrbsc.org/gfx/genedoc/ebinet.htm) multiple sequence alignment tool was used to generate the multiple protein sequence alignment of B-Box and CCT domains of PaCOL proteins together with *Arabidopsis* and rice PaCOL proteins. The conserved motifs were analyzed using Multiple EM for Motif Elicitation (MEME) web tool (https://meme-suite.org/meme/) following the parameters: maximum number of motifs 10, minimum width 6, and maximum width 50. NCBI BLAST search tool (https://blast.ncbi.nlm.nih.gov/Blast.cgi) was used to identify the query coverage percentage and identity of each gene. These query coverage percentage and identity of each gene were employed to find out any segmental or tandem duplication of petunia *COL* genes according to Kong et al. (2013) and Wang et al. (2010), respectively [40, 41].

### Phylogenetic analysis

Phylogenetic analysis of *Petunia axillaris*, *Arabidopsis*, rice, maize, tomato and *Physcomitrella patens* COL proteins was conducted to determine evolutionary relationships of COL family proteins among the six plant species. Phylogenetic analysis of *Petunia inflata* was presented in Figure S4. In order to construct phylogenetic tree we selected Arabidopsis, rice, maize COL proteins as representative species because COL family has been well characterized in these plant species. Besides, *Arabidopsis* is model dicot plant; rice and maize are model monocot plant for comparative research of gene function in plant biology. Tomato has been used in the phylogenetic analysis because tomato and petunia belong to the same *Solanaceae* family and have close evolutionary relation. *Physcomitrella patens* was used as primitive origin proteins. The full length COL protein sequences (15) of petunia were aligned with those of Arabidopsis (17), rice (17), maize (19), *Physcomitrella patens* (17) and tomato (13) using ClustalW program (build-in MEGA 7.0). The phylogenetic analysis was conducted using the maximum likelihood method in MEGA 7.0 software [42]. All members of the COL family proteins in *A. thaliana* were downloaded from the TAIR database, whereas those proteins maize, tomato were acquired from the NCBI (https://www.ncbi.nlm.nih.gov/) and Sol Genomics databases (https://www.sgn.cornell.edu/) and Phytozome database (https://phytozome.jgi.doe.gov/pz/portal.html), respectively presented in Table S7.

### Identification of cis-acting elements and putative biological functions

PlantCARE web-based tool (http://bioinformatics.psb.ugent.be/webtools/plantcare/html/) was employed to speculate the putative *cis*-regulatory elements using 2000-bp upstream region of the initiation codon “ATG” [43]. The molecular and biological functions of petunia *COL* genes were analyzed using Blast2GO (https://www.blast2go.com/) web tool [44].

### Plant materials and growth conditions

*Seeds of Petunia. hybrida* cv. Mirage Rose were germinated in potted soil in a growth chamber at 25 °C day/20 °C night, 16-h light/8-h dark photoperiod, with relative humidity ranging from 60 to 70%, and a light intensity of 300 μmol m^*−* 2^ s^*−* 1^. Five-week old seedlings were transferred to a greenhouse to allow further growth in greenhouse at 20 *±* 2 °C temperature with a relative humidity ranging from 65 to 80%. Different vegetative plant samples e.g. seedlings at 3 weeks after sowing, leaves, stem buds and root samples at 4 week after sowing were collected from the seedlings for tissue specific expression analysis. The remaining seedlings were transferred to a greenhouse to allow their further growth at 20 *±* 2 °C temperature with 65 to 80% relative humidity. Reproductive tissues of petunia at the different developmental stages e.g., (i) flower bud (petal length < 0.5 cm, Stage1), (ii) full blooming flower and (iii) senescing flower were harvested from petunia for analyzing the tissue-specific expression patterns of *PaCOL* genes. Three biological replicates were sampled from each treatment. Flower buds and full blooming flowers were collected at the anthesis stage [45]. The collected organ samples were frozen immediately in liquid nitrogen and stored at − 80 °C.

### Stress treatments

Leaves from 35-days old seedlings were selected for stress treatment. The seedlings were incubated at 4 °C in a growth cabinet for cold treatments for 24 h. The COL proteins play a central role in photoperiodic flowering control of plants by mediating the input signals of temperature and light [46]. Therefore, leaves from five-weeks old petunia seedlings were exposed to two levels of temperature (37 °C and 41 °C) to test whether they could be induced by heat stress (Fig. 5b).

Seedlings were incubated in a growth cabinet at 37 °C and 41 °C respectively for 24 h to impose heat treatment. To impose ABA treatment 100 μM ABA was sprayed over the seedlings and covered those for 24 h to facilitate ABA absorbance. The seedlings were gently pulled out from the soil and placed on a paper towel for 24 h under normal greenhouse conditions (20 *±* 2 °C temperature with a relative humidity ranging from 65 to 80% and 14 h light/10 h dark) to induce drought treatment. The seedlings were kept in 200 mM NaCl with Hoagland solution for 24 h to impose salinity treatment. For waterlogging treatment the seedlings were put in a big bowel filled up with water up to the collar region for 48 h. Plants grown in pot soil under normal conditions at 25 °C were sampled as the ‘non-treated control’ for all treatments. Treated leaf samples were collected at 0 h (control), 1, 3, 9 and 24 h after the treatment except waterlogging stress. Leaves from the waterlogged treated samples were sampled at 0 h (control), 3, 9, 24 and 48 h after the treatment. In case of ABA and NaCl treatments water was sprayed instead of ABA and NaCl on plant leaves to collect mock sample. For all samples three biological replicates were conducted. All collected samples were immediately frozen in liquid nitrogen and preserved at − 80 °C for further analysis.

### RNA preparation, cDNA synthesis, RT-PCR and qRT-PCR analyses

The Qiagen RNeasy Mini Kit (QIAGEN, Hilden, Germany) was used to extract the total RNA according to the manufacturer’s protocol. Gel electrophoresis and a NanoDrop 1000 spectrophotometer (NanoDrop Technologies, Wilmington, DE, USA) were utilized to determine the quality and quantity of each RNA sample. Superscript First-Strand cDNA Synthesis System (Invitrogen, Carlsbad, CA, USA) was used to synthesize the cDNA reactions (1 μg of total RNA per reaction used as a template) according to the manufacturer’s instructions. RT-PCR of petunia samples was performed using an AMV one step RT-PCR kit (Takara, Shiga, Japan). Gene-specific primers used in RT-PCR analysis were listed in Table S1. Primer3 software (http://frodo.wi.mit.edu/primer3/input.htm) was used to design the primers. cDNA samples of different organs were used as DNA template. The 20 ml PCR mixture contained 1 mL cDNA sample, 8 mL Emerald PCR Master Mix (Takara, Shiga, Japan), 1 mL each of forward and reverse primers and 9 mL double-distilled H_2_O. The PCR conditions were as follows: pre-denaturation for 5 min at 94 °C, amplification for 30 cycles at 94 °C for 30 s, annealing for 30 s at 58 °C and extension for 1 min at 72 °C, with a final extension at 72 °C for 5 min. The Quantitative real-time PCR (qRT-PCR) was carried out using the LightCycler96 (Roche, Mannheim, Germany) thermal cycler according to the manufacturer’s protocol. A total volume of 10 μL reaction mixture containing 1 μL of 50 ng cDNA, 2 μL forward and reverse primers of 10 pmol concentration, 5 μL iTaqTM SYBR® Green PCR kit (PCRBIOSYSTEMS, London, UK) and 2 μL double distilled water was prepared for qRT-PCR analysis. The PCR condition was set as: primary denaturation at 95 °C for 300 s followed by 40 amplification cycles at 94 °C for 10 s, annealing at 58 °C for 10 s and extension at 72 °C for 15 s. The melting temperature was set to 95 °C for 10 s, 65 °C for 60 s and 97 °C for 1 s to ensure there was no primer-dimer formation. The relative expression levels of the *PaCOL* genes were normalized against the house-keeping gene, *Elongation factor 1a* (*EF1a*: accession no. LOC101268350) [47] and the relative amount of the amplified product was calculated following the 2^−ΔΔCt^ method [48]. Seedling was harvested first and that represented both shoots and roots. That’s why seedlings were taken as a calibrator Whereas the leaf samples were collected at 0 h after treatment was the calibrator for abiotic stress and hormone treatments [49]. Primers used for qRT-PCR based gene expression analysis were designed using Primer3 software (http://frodo.wi.mit.edu/primer3/input.htm) software (Table S1).

### Computation of Ka/Ks values

OrthoMCL software (v2.0.3) (Li 2003) was utilized for searching the paralogous genes in petunia considering the E-value 1e^− 5^, and the alignment with a match cut-off value more than 50 [50]. The evolutionary constraint (Ka/Ks) between paralogous pairs of genes of petunia were calculated following the method developed by Nei and Gojobori (http://cbb.big.ac.cn/software) [51]. The divergence time was computed using the formula T = Ks/2R, Mya (millions of years), where R is the constant for dicotyledonous plants of 1.5 × 10^− 8^ substitutions per site per year, and Ks is the synonymous substitution rate per site [52].

### Statistical analysis

A one-way analysis of variance (ANOVA) was performed with MINITAB 18 statistical software (Minitab Inc., State College, PA, USA) to perceive the statistical significance in relative expression levels of genes among organ samples and also between treatments (control versus stress). Tukey’s pairwise comparison was conducted for the mean separation of expression level of the genes.

## Results

### Identification of COL family proteins in petunia species

A total of 17 petunia COL genes were identified and the existence of the conserved domains was checked using InterProScan and SMART tools to confirm their reliability. The results revealed that 15 out of 17 genes contained both BBOX and CCT domain. The genes were named from *PaCOL1* to *PaCOL15* following the naming convention (Table 1). Two candidate genes were excluded because of their incomplete domain structure. Bioinformatics analyses showed that the length of PaCOL proteins ranged from 336 to 467 amino acids (aa), and their molecular weight varied from 37.53 to 51.39 kDa with a pI ranged between 4.97 and 6.35. Inferred from the pI structures, all PaCOL proteins were acidic proteins (pI < 7), and the GRAVY indicated that all these proteins were hydrophilic; since the GRAVY values of all were negative (Table 1). Moreover, all of the 15 PaCOL proteins predicted to be located in nucleus (Table 1).

**Table 1:** Sequence analysis of petunia COL proteins identified in *Petunia axillaris* genome

Gene name	Locus ID	ORF (bp)	Strand	No. of introns	Proteins						
					Length (aa)	Domain Start-end (aa)		MW (kDa)	p^**I**^	GRAVY	Subcellular localization
						BBOX	CCT				
*Pa*COL1	Peaxi162Scf00007g01220	1224	(+)	03	407	2–47	351–394	44.08	5.74	−0.400	Nuclear
*Pa*COL2	Peaxi162Scf00012g03214	1302	(−)	03	434	2–47	392–422	48.32	5.99	−0.568	Nuclear
*Pa*COL3	Peaxi162Scf00015g00927	1224	(+)	01	407	17–61	350–392	46.41	4.97	−0.777	Nuclear
*Pa*COL4	Peaxi162Scf00020g01929	1149	(+)	02	382	52–99,12–56	313–355	42.83	5.69	−0.790	Nuclear
*Pa*COL5	Peaxi162Scf00045g01824	1011	(+)	01	336	43–90, 2–47	273–316	37.53	5.73	−0.517	Nuclear
*Pa*COL6	Peaxi162Scf00047g02226	1242	(−)	03	413	4–47, 47–77	356–399	45.32	5.42	−0.629	Nuclear
*Pa*COL7	Peaxi162Scf00067g01119	1152	(−)	03	383	18–59, 62–97	332–375	43.11	5.57	−0.769	Nuclear
*Pa*COL8	Peaxi162Scf00128g01749	1404	(−)	03	467	9–43, 56–99	419–462	51.39	6.24	−0.719	Nuclear
*Pa*COL9	Peaxi162Scf00359g00213	1239	(−)	03	412	5–47, 47–78	355–398	45.12	5.09	−0.575	Nuclear
*Pa*COL10	Peaxi162Scf00382g00067	1326	(+)	01	441	17–61	386–428	49.88	5.53	−0.711	Nuclear
*Pa*COL11	Peaxi162Scf00416g00214	1389	(+)	03	462	5–47, 43–72	406–449	51.08	5.58	−0.468	Nuclear
*Pa*COL12	Peaxi162Scf00459g00510	1071	(−)	01	356	51–98, 12–55	293–335	39.45	5.76	−0.522	Nuclear
*Pa*COL13	Peaxi162Scf00581g00039	1242	(−)	03	413	4–47, 44–90	359–399	45.34	5.91	−0.616	Nuclear
*Pa*COL14	Peaxi162Scf00942g00122	1140	(+)	01	379	58–105, 21–62	305–347	41.59	6.35	−0.301	Nuclear
*Pa*COL15	Peaxi162Scf01346g00018	1218	(−)	01	405	17–61	351–393	46.40	5.44	−0.832	Nuclear

### Sequences alignment and phylogenetic analysis

**Fig. 1:**
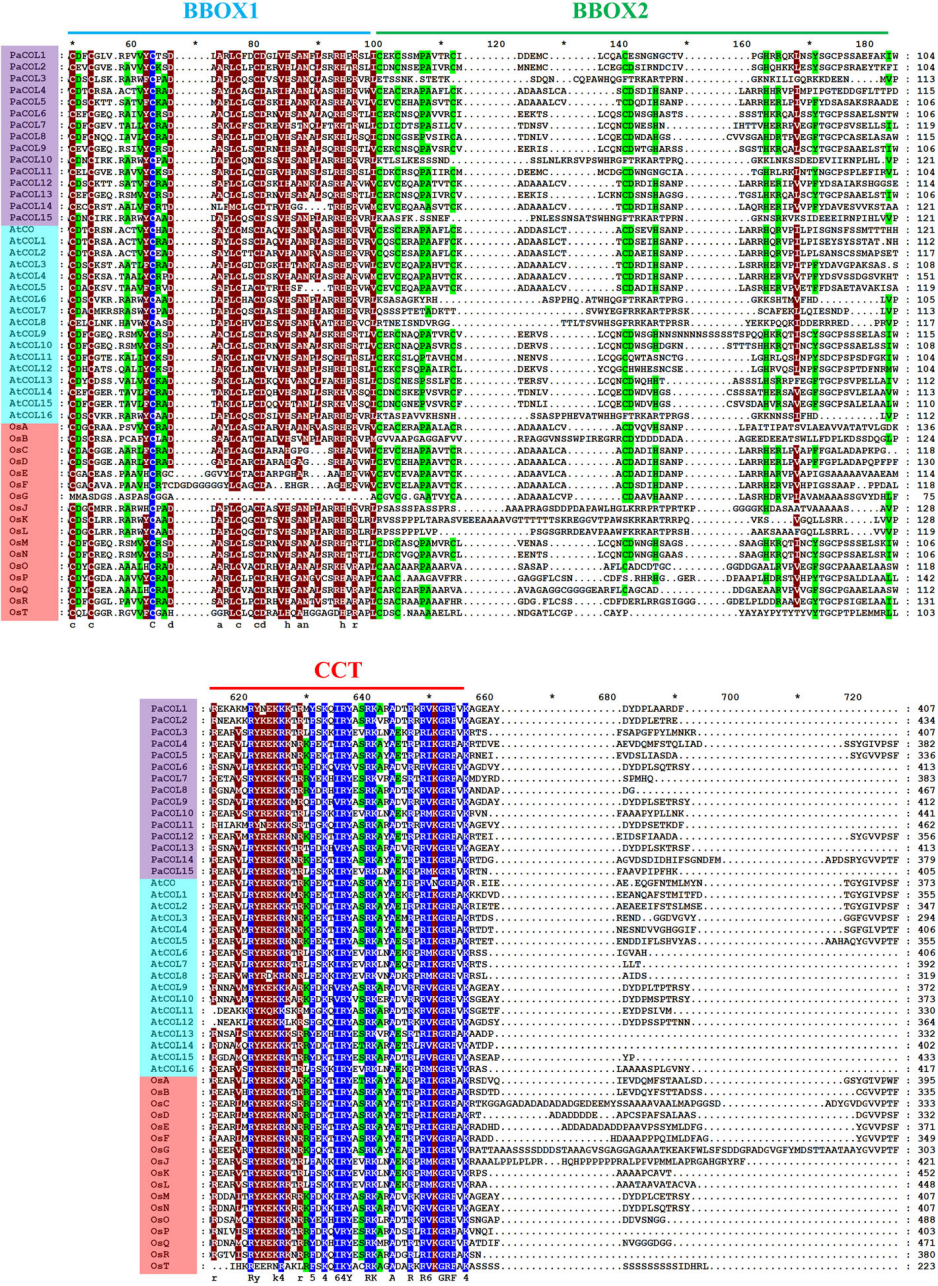
Sequence alignment of COL proteins from petunia, *Arabidopsis* and rice. Each letter represents one amino acid, and the left column corresponds to the name of the gene. The BBOX1 and BBOX2 domains are indicated by the blue and green line and CCT domains are indicated by the red line respectively. The red region indicates residues conserved only in the BBOX1 domain, the green region indicates residues conserved in the BBOX2 domain and the indigo region indicates residues conserved in the CCT domain of CO-like proteins

Multiple alignment analysis of putative PaCOL proteins containing BBOX and CCT domains revealed that these two domains were highly conserved despite their difference in sequence lengths and properties (Fig. 1, Figure S1). Eight proteins (PaCOL4, PaCOL5, PaCOL6, PaCOL8 PaCOL9, PaCOL12, PaCOL13 and PaCOL14) contained one CCT domain and two BBOX (Fig. 1 and Figures S1 and S2), two proteins contained (PaCOL1, PaCOL2) one CCT domain and one BBOX domain, and five proteins (PaCOL3, PaCOL7, PaCOL10, PaCOL11 and PaCOL15) contained one CCT domain, one co-like BBOX domain and one diverged zinc finger domain (Fig. 1, Figure S2). COOH-like amino acid residues were observed in the C-terminal region of petunia COL proteins (Fig. 1). The results are consistent with the previous findings where COL genes were divided into three groups based on the number and type of BBOX domain [6]. The BBOX is one type of conserved zinc fingers domain characterized by C-X2-C-X8-C-X7-C-X2-C-X4-H-X8-H patterns. The similar amino acid patterns were found in the all PaCOL proteins.

**Fig. 2:**
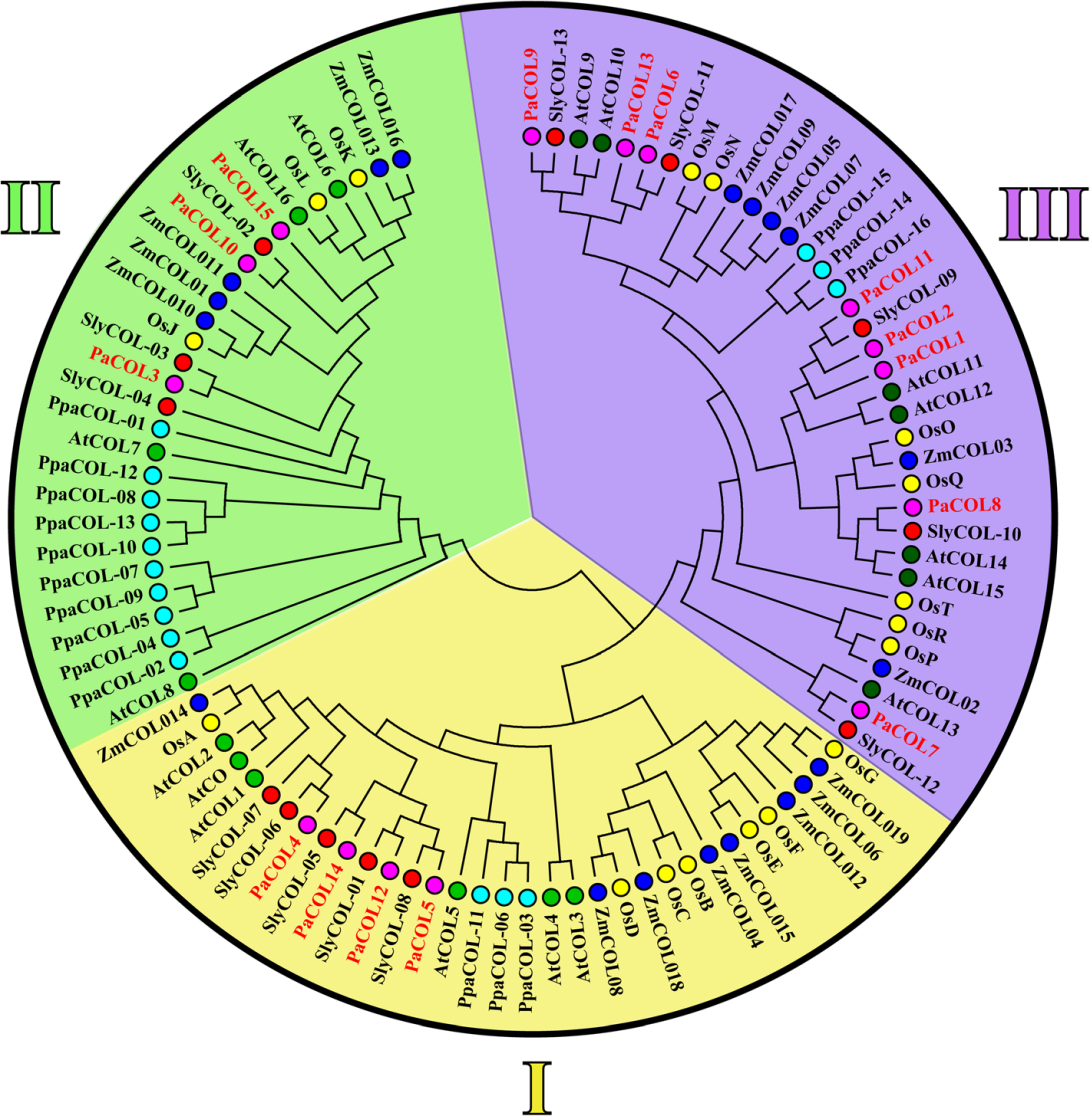
Phylogenetic analysis of the COL proteins from different plant species. The phylogenetic tree was established with entire protein sequences from the above plant species by MEGA 7.0 software using the Maximum Likelihood method. The numbers on the branches indicate bootstrap support values from 1000 replications. The scale represents the units of the number of amino acid substitutions per site

A comparative phylogenetic analysis was performed to provide an insight into the evolution of *COL* genes in different species (Fig. 2). The COL proteins from different species were clustered into three groups, designated as I-III which was generally consistent with previous reports [6]. Group I members had one CCT domain and two zinc finger B-boxes domain whereas groups II and groups III members had only one B-box domain along with one CCT domain (Fig. 2). Groups III members (except PaCOL1 and PaCOL2) had an additional diverged zinc finger domain besides B-box and CCT domain (Fig. 2). Group III had the largest number of *PaCOL* genes (7 out of 15) while Group I had 5 and group II had 3 *PaCOL* genes respectively (Fig. 2). As shown in Fig. 2, the *PaCOL* genes always clustered close to tomato *COL* genes and displayed high sequence similarities. Moreover, the *COL* genes from monocots (rice and maize) were closely grouped in the phylogenetic tree. The group II was the smallest among the three groups and contained maximum number of COL members from *Physcomitrella patens* (Fig. 2). Three, three and ten *PpaCOL* genes in *Physcomitrella patens* were classified into Groups I, II and III, respectively, consistent with previous reports [50].

### Gene structure and motif composition of *PaCOL* genes

**Fig. 3:**
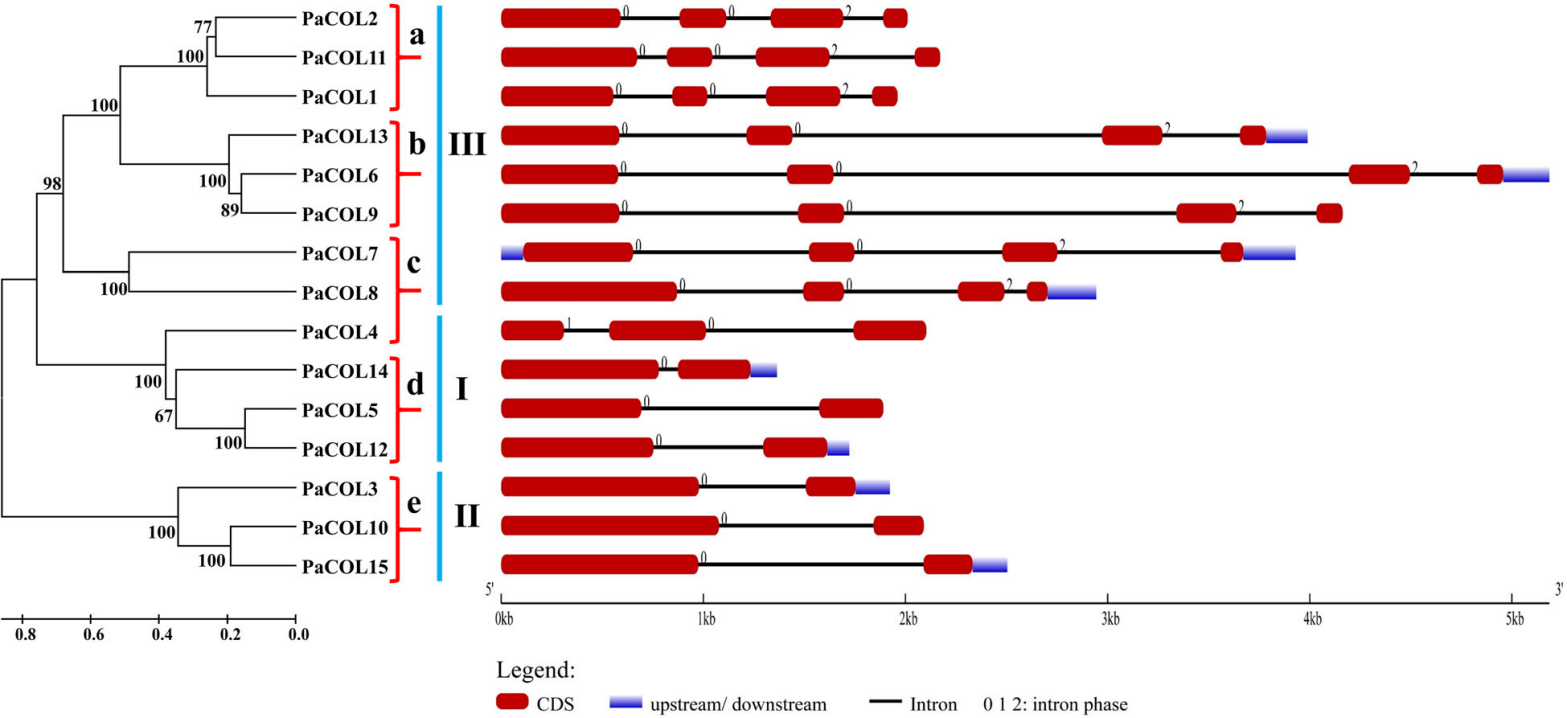
Analysis of gene structure in *PaCOL* genes in light of the phylogenetic relationship. Red boxes, blue boxes, and black lines indicate exons, upstream/downstream, and introns, respectively

The number of introns ranged from 1 to 3 and exons ranged from 2 to 4 respectively among the *PaCOL* genes (Fig. 3). As shown in Figs. 2 and 3, PaCOL proteins were classified into three distinct groups and a strong correlation existed between gene structure and phylogeny. Number of exon/intron and their pattern of distribution were also similar among the members of an individual cluster within a group. For example, *PaCOL1, PaCOL2, PaCOL11* of cluster a*, PaCOL6, PaCOL9, PaCOL13* of cluster b under group III and *PaCOL3, PaCOL10, PaCOL15* of cluster e under group II had almost similar number of exon-intron and pattern of distribution and they exhibited a closer evolutionary relationship (Figs. 2 and 3). It was also found that *PaCOL1*, *PaCOL2*, *PaCOL4* and *PaCOL11* genes had no upstream/downstream regions.

Maximum number of introns was found to be located on phase 0 followed by phase 2. Only one intron was found to be in phase 1 (Fig. 3). The first and the last intron phase of all the intron-containing *PaCOL* genes under Group III were 0 and 2, respectively (Fig. 3). Except for the *PaCOL4* the intron phase of all the *PaCOL* genes under Group I and Group II were 0 (Fig. 3). The first and the last intron phase of *PaCOL4* in Group I was 1 and 0, respectively (Fig. 3).

MEME motif search identified 10 conserved motifs; motif 1 represented as CCT domain and motif 2 and 3 represented as BBOX 1 and 2 domains respectively, where others 7 motifs had no functional annotations (Figure S3). Despite the discrepant protein sequences the members those clustered together in the phylogenetic tree had similar motif features (Fig. 2, Figure S3).

### Putative *cis*-acting elements and functional analysis of *PaCOL* genes

*cis*-acting regulatory elements potentially associated with the regulation of gene expression under various abiotic and biotic stresses [53]. A total of 35 putative *cis*-acting regulatory elements were identified including various stress-responsive *cis*-acting regulatory elements in the putative promoter regions of *PaCOL* genes (Table S2). For example, ABA-responsive elements (ABRE), SA-responsive elements (TCA-element), ethylene-responsive elements (ERE), low-temperature response (LTR) elements, defense and stress responsiveness (TC-rich repeats, ARE and MBS) elements were detected in the promoter regions of *PaCOL* genes. Additionally, wound-responsive elements (WUN-motif), MeJA-responsive elements (TGACG-motif and CGTCA-motif) were also detected in some *PaCOL* genes. In particular, *cis*- acting elements involved in MeJA-mediated responses, including the MeJA-responsive elements (TGACG-motif and CGTCA-motif), as well as the wound-responsive elements (WUN-motif) and the MYC-binding site (G-box) were also discovered (Table S2). Moreover, the putative biological function analysis revealed that the *PaCOL* genes were likely to be involved in some similar and common groups including molecular Function, biological function and cellular components (Table S3). For example, molecular function include, GO:0008270: Zinc ion binding, transition metal ion binding, metal ion binding, cation binding, ion binding; GO: 044212: DNA binding, nucleic acid binding, organic cyclic compound binding, heterocyclic compound binding; biological function include, GO:0006355: regulation of transcription, Go:0007623: circadian rhythm, GO: 0009909: regulation of flower Development, GO: 0009266: response to temperature, GO:0009651: response to salt stress, GO: 0006950: response to stress and cellular components, GO: 0005730: nucleous (Table S3).

### Calculation of Ka/Ks ratios

Selection mechanisms of *COL* genes during evolution were disclosed using the non-synonymous (amino acid-replacing, Ka) and synonymous (Ks) substitution rates with a Ka/ Ks > 1 specifying positive selection, Ka/Ks < 1 indicating purifying (negative) selection, and a Ka/Ks close to 1 signifying a neutral mutation [54]. The Ka, Ks and divergence time among the paralogous gene-pairs were shown in Table S4. All of the *PaCOL* paralogous gene-pairs accounted for Ka/Ks ratios less than 1 indicated a strong purifying/negative selection pressure in these genes in the evolutionary processes. The divergence time estimated to occur between 20.2 (Ks = 0.6061) to 43.82 (Ks = 1.3146) million years ago (Mya) (Table S4).

### The expression analysis of *PaCOL* genes in different tissues

**Fig. 4:**
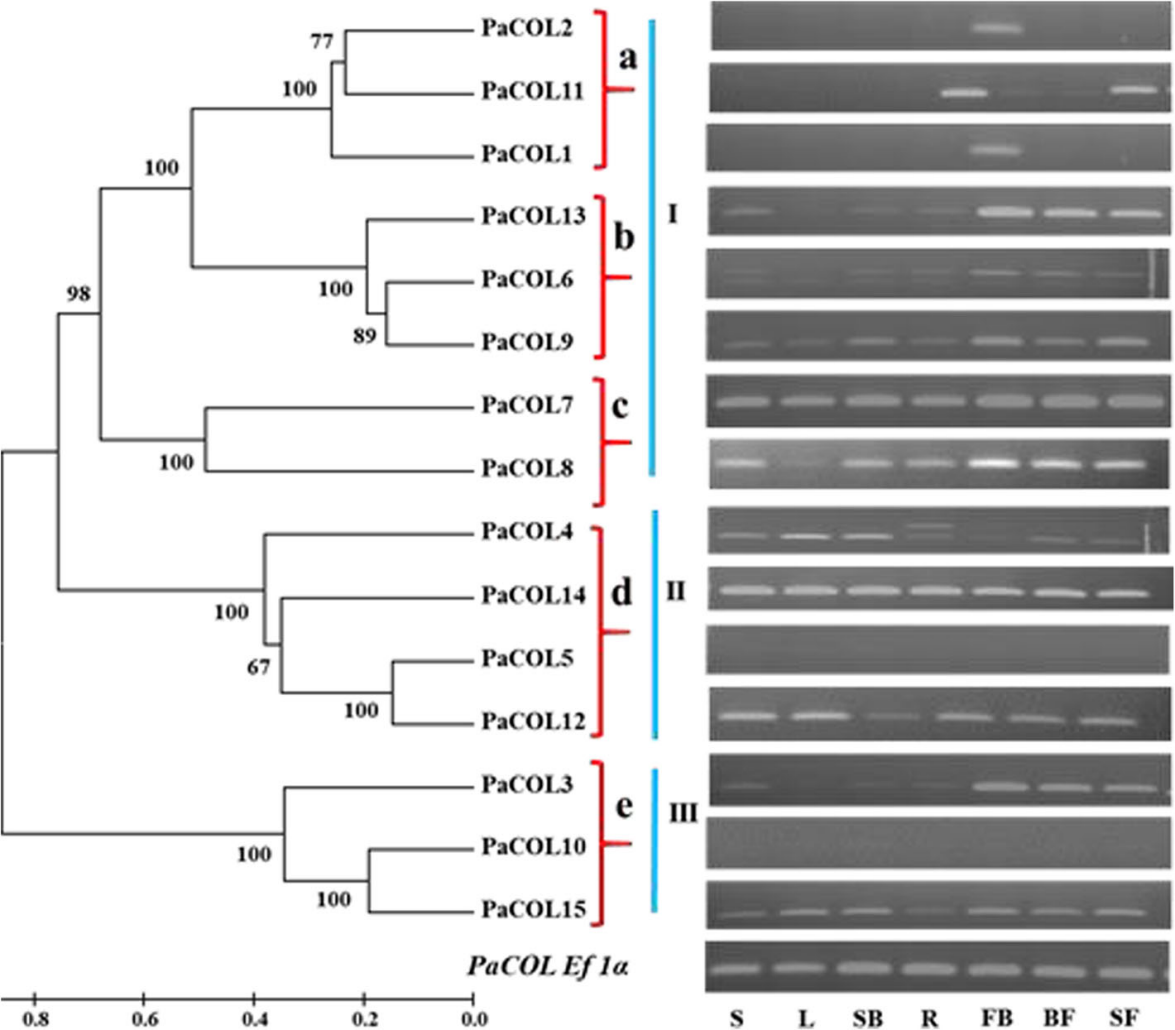
The RT-PCR expression patterns of *PaCOL* genes in different petunia tissues (S-seedling (3 week old), L - leaves, SB-stem bud and R-root from the 4 weeks old seedlings, FB-flower buds (from 6 weeks plants), BF- full blooming flower (from 8 weeks old plants), SF- senescencing flower). The level of expression was nor malized to petunia *EF1α* gene

The expression profiles of *PaCOL* genes in different tissues, namely– seedlings (3 weeks old), leaves, stem bud, root, flower bud, full blooming flower and senescing flower were diverse. Some genes were constitutively expressed in these seven tissues, namely *PaCOL3*, *PaCOL6, PaCOL7, PaCOL9*, *PaCOL12, PaCOL14* and *PaCOL15* (Fig. 4). Some genes had specific expression characteristics to some extent in a particular tissue, for example– *PaCOL1, PaCOL2* and *PaCOL11* were strongly expressed in flower buds compared to the other tissues (Fig. 4). *PaCOL13* and *PaCOL15* were highly expressed in different developmental stages of flower (flower bud, full blooming flower and senescence flower). The transcript levels of some genes, namely- *PaCOL3, PaCOL6, PaCOL12, PaCOL13,* and *PaCOL15* were weakly expressed in root tissues relative to other organs (Fig. 4). When combined with our phylogenetic investigation (Fig. 3) and expression analysis (Fig. 4) it has been noticed that several gene pairs belonging to the same phylogenetic groups shared almost similar expression patterns in the tested organs and displayed a positive correlation (Figs. 3 and 4). So based on the expression pattern, the *PaCOL* genes could roughly be divided into five clades (a-e) (Figs. 3 and 4). All genes of clade a, b, c belonged to Group III, and all genes of clade d and e belonged to group I and group II, respectively (Figs. 3 and 4). Several paralogous gene-pairs clustered together in the same clade shared similar expression patterns in different organs examined (Figs. 3 and 4). For example, *PaCOL1, PaCOL2, PaCOL11*, of the ‘clade a’ were expressed at the highest levels in the flower buds; *PaCOL6, PaCOL9, PaCOL13* of the ‘clade b’ were most highly expressed in flower buds, full blooming flowers and senescing flowers respectively (Fig. 4).

### The expression analysis of *PaCOL* genes under different stresses

#### Heat stress

**Fig. 5:**
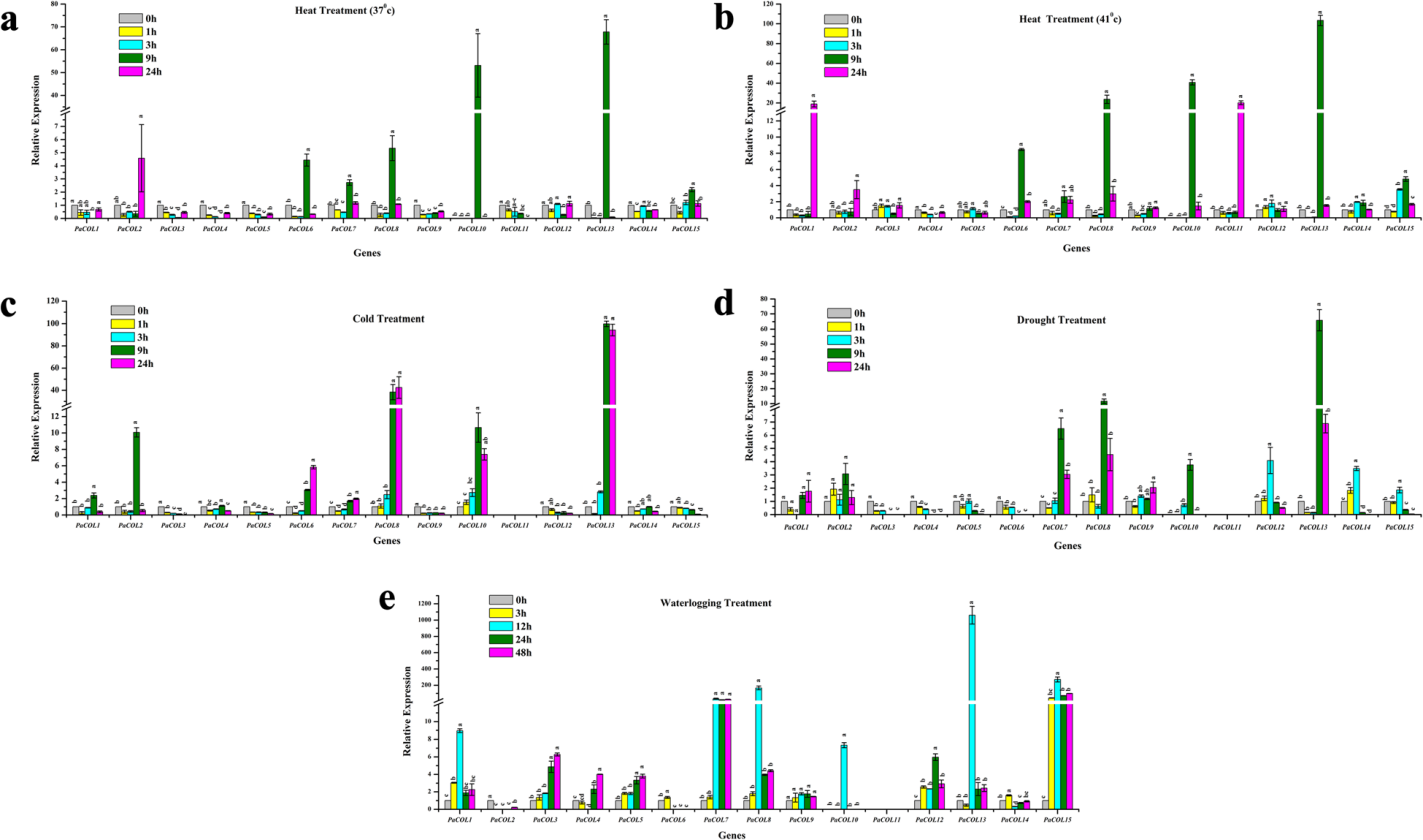
**a**-**e** Expression profiles of 15 *PaCOL* genes in leaf samples under heat (37 °C and 41 °C), cold, drought, waterlogging treatment. The data represented the expression levels of *PaCOL* genes at 0 h, 1 h, 3 h and 24 h after the heat, cold, drought treatments and 0 h, 3 h, 12 h, 24 h and 48 h after waterlogging treatment. Samples at 0 h refer the untreated plants (control plants) under normal conditions. The results were calculated via the 2^−ΔΔCt^ method, and the reference gene (*EF1α*) was used to correct the expression level of target genes. The expression level of 0 h was set as 1. The data were presented as the means of three biological replicates and three technical replicates, and the error bars represented the standard error of the means. Different letters above the bars indicate significant differences (*p* < 0.05) among treatments

The COL proteins play a central role in photoperiodic flowering control of plants by mediating the input signals of temperature and light (Ke 2020). Therefore, leaves from five-weeks old petunia seedlings were exposed to two levels of temperature (37 °C and 41 °C) to test whether they could be induced by heat stress (Fig. 5a, b).

The expression level of two genes *PaCOL10* and *PaCOL13* dramatically increased by 40- to 100- fold at 9 h after the treatment both at 37 °C and 41 °C temperature compared to control (Fig. 5b). Besides, the relative expression level of *PaCOL6, PaCOL8* genes increased by 8- and 20-fold, respectively at 9 h after the treatment and that of *PaCOL11* increased by 20-fold at 24 h after the treatment under 41 °C temperature (Fig. 5b). At 37 °C temperature– the expression of *PaCOL6, PaCOL7* and *PaCOL8* genes increased from 3- to 5-fold at 9 h after treatment compared to control (Fig. 5). The other genes had no remarkable expression at high temperature treatment (Fig. 5b).

#### Cold treatment

Six out of fifteen *COL genes* (*PaCOL2, PaCOL6, PaCOL8, PaCOL10* and *PaCOL13)* were induced by cold treatment (Fig. 5c).

Among those, *PaCOL8* and *PaCOL13* genes were highly expressed by 40- and 100-fold at 9 h and 24 h after the treatment, respectively, compared to control (Fig. 5c). *PaCOL2* was also induced by the cold stress and the relative expression level of this gene peaked (~ 10 fold) at 9 h after the treatment compared to control (Fig. 5c). *PaCOL6,* and *PaCOL8* genes were up-regulated from 1.8- to 22.5-fold at 9 h and 24 h after the treatment relative to the control (Fig. 5c). The remaining genes were not significantly induced by the cold treatment (Fig. 5c).

#### Drought treatment

Among the 15 genes the expression level of *PaCOL7, PaCOL8* and *PaCOL13* were highly induced at 9 h and 24 h (3- to 70- fold respectively) after drought treatment (Fig. 5d), while that of *PaCOL10, PaCOL12* and *PaCOL14* were induced (from 2- to 4- fold) only at 9 h and 3 h after treatment respectively compared to control (Fig. 5d). The remaining genes exhibited no remarkable expression after drought treatment (Fig. 5d).

#### Waterlogging treatment

Four genes out of 15 displayed diverse expression pattern after waterlogging treatment (Fig. 5e). *PaCOL8* were induced by 200-fold and *PaCOL13* were strongly induced by around 1200-fold only at 12 h after treatment compared to control (Fig. 5e). Expression of *PaCOL7* gene was induced at 12, 24 and 48 h around 100- fold and the expression of *PaCOL15* was induced at 3, 12, 24 and 48 h after treatment by 150-, 200-, 150- and 150- fold, respectively (Fig. 5e).

#### Salinity treatment

**Fig. 6:**
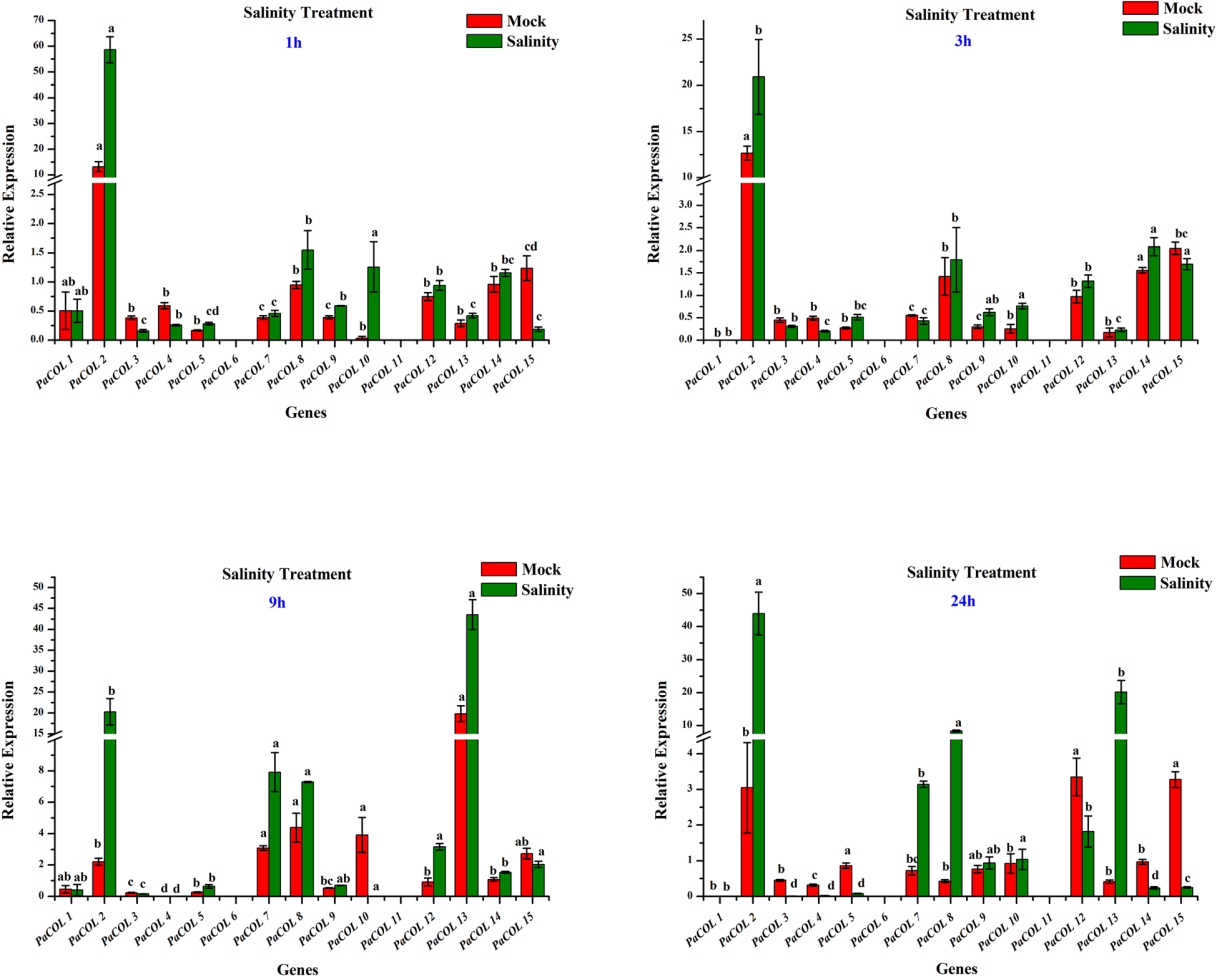
Expression profiles of 15 *PaCOL* genes in leaf samples under salinity treatment. The data represented the expression levels of *PaCOL* genes at 0 h, 1 h, 3 h and 24 h after salinity treatment. Samples spraying with water at 0 h, 1 h, 3 h and 24 h refer the mock plants. The results were calculated via the 2^−ΔΔCt^ method, and the reference gene (*EF1α*) was used to correct the expression level of target genes. The expression level of 0 h was set as 1. The data were presented as the means of three biological replicates and three technical replicates, and the error bars represented the standard error of the means. Different letters above the bars indicate significant differences (*p* < 0.05) among treatments

Notably 7 out of 15 genes– *PaCOL2, PaCOL7, PaCOL8, PaCOL10, PaCOL12, PaCOL13,* and *PaCOL14* showed significant and differential expression at different time point under salinity stress compared to mock (Fig. 6). Three genes *PaCOL2, PaCOL8,* and *PaCOL13* were highly up-regulated where *PaCOL2* induced by 40- fold, *PaCOL8* induced by 2 to 10- fold and *PaCOL13* induced by 20- to 40- fold throughout the stress period compared to mock (Fig. 6). By contrast, three other genes namely *PaCOL3, PaCOL4,* and *PaCOL15* were down-regulated in saline-treated samples compared to the mock. The expression of *PaCOL7* and *PaCOL8* initially showed no response but those were up-regulated at 9 h and 24 h after treatment (Fig. 6). The remaining genes showed little or no change in response to salinity stress (Fig. 6).

#### ABA treatment

**Fig. 7:**
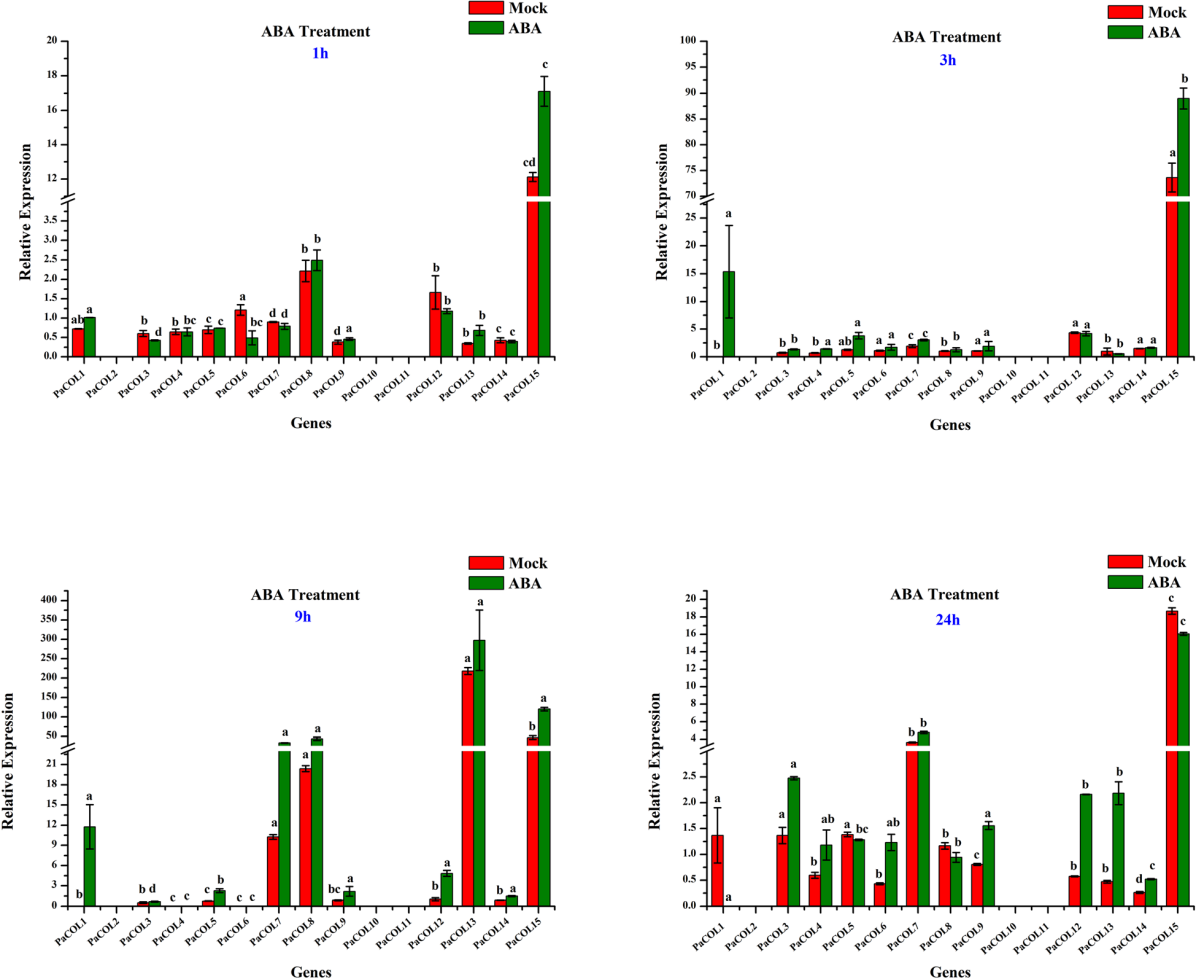
Expression profiles of 15 *PaCOL* genes in leaf samples under ABA treatments. The data represented the expression levels of *PaCOL* genes at 0 h, 1 h, 3 h and 24 h after ABA treatment. Samples spraying with water at 0 h, 1 h, 3 h and 24 h refer the mock plants. The results were calculated via the 2^−ΔΔCt^ method, and the reference gene (*EF1α*) was used to correct the expression level of target genes. The expression level of 0 h was set as 1. The data were presented as the means of three biological replicates and three technical replicates, and the error bars represented the standard error of the means. Different letters above the bars indicate significant differences (*p* < 0.05) among treatments

Eight *PaCOL* genes out of 15 were significantly induced by ABA treatment (Fig. 7). *PaCOL15* displayed up-regulated expression under all treatment period compared to mock whereas, *PaCOL12, PaCOL13, PaCOL14* showed up-regulated response at 9 h and 24 h after treatment compared to mock (Fig. 7). Another six *PaCOL* genes *PaCOL1, PaCOL3, PaCOL4, PaCOL5, PaCOL7,* and *PaCOL8* showed variable change in response to ABA application compared to mock (Fig. 7).

## Discussion

*COL* genes are widely existing in plant kingdom and likely to be involved in controlling flowering time. A few studies in *Arabidopsis*, rice, maize reported that the *COL* gene family regulate plant development and improve plant’s resistance to abiotic stress [6, 55]. However, the evolutionary and expression analyses of the *COL* genes have not been reported to date in any solanaceous species. In the present study, a genome-wide survey of the *COL* gene family in petunia identified a total of 15 genes taking both B-box and CCT domain on protein sequences into account [6]. Consistent with the names of corresponding *A. thaliana* and rice genes– *COL* genes of petunia were renamed as *PaCOL1-PaCOL15*. Despite the wide differences in overall genome size e.g., 164 Mbp in Arabidopsis, 441 Mbp in rice, 950 Mbp in tomato, 2300 *Mbp* in maize *and 1260 Mbp in petunia,* the number of *COL* genes varied between 15 and 19 in *Arabidopsis,* rice, maize and petunia [56, 57]. The results indicated that genetic constitution of the *COL* genes is quite conserved in those plant species during the evolutionary process [58]. However, in contrast to tomato [13], Petunia appears to contain a little larger number of COL genes (15 vs. 13), suggesting possible duplication events of COL genes in *Petunia* genome or rapid loss of some members in tomato. The physical and chemical properties (e.g., MW, the number of amino acids, isoelectric points) of 15 PaCOL proteins varied widely in petunia indicating that *COL* genes change their properties during the process of evolution.

The protein structural framework is important for the prediction of perfect functioning of proteins. The conserved protein motifs and the gene structure of PaCOL showed a similar trend as those described earlier with known COL homologs involved in photoperiod-responsive plant species suggesting the possible functional conservation during the evolution of a wide range of plant species (Fig. 3, Figure S3) [59]. Moreover, consistent with the AtCOLs, petunia COLs are clustered into three groups (Fig. 2) [6]. All of the three phylogenetic groups contained the *COL* genes from both monocots and dicots and the existence of monocots and dicots *COL* genes in a common clade suggesting that *COL* gene family originated and diversified prior to the divergence of monocotyledons and dicotyledons [58]. The *COL* genes of petunia are more closely allied with tomato, where those of rice are more closely allied with maize compared to *Arabidopsis* representatives indicating the species specific evolutionary relationship between monocotyledons and dicotyledons [58]. The phylogenetic tree clearly depicted the evolutionary relatedness between the *COL* genes of different plant species which is consistent with a previous reports of Ke et al. 2020 and Qin et al. 2018 [46, 59]. Many genes those were clustered in a clade with a high bootstrap value showed similar intron number and motif distribution (Figs. 2 and 3). For example, PaCOL1, PaCOL2, PaCOL11 and PaCOL6 and PaCOL9 in the ‘group III’ shared same number of introns, motifs and the arrangement of motif. However, different members belonging to different phylogenetic groups showed variations in exon-intron and motif distributions. This apparent association among the phylogenetic classes, arrangement of motifs and number of introns indicated that these genes have a conserved structural pattern within the same phylogenetic group that favours their functional classification.

Intron phase distribution is conserved and non-uniform during the evolutionary process of plant species showing an obvious distribution pattern of phase-0 > phase-1 > phase-2 [60]. Introns can be located in one of three phases: − 0, − 1, and − 2 where 0 phase located before the first nucleotide of a codon and − 1, and − 2 phases located after the first, and after the second nucleotide of a codon, respectively [60]. The phase 0 introns were the most frequently occurring while phase 2 introns occurred the least frequently [60]. However, the intron phase distributions predicted in *PaCOL* genes showed contradictory phase distribution pattern in which the distribution of phase-2 introns is larger than that of phase-1 introns (Fig. 3). Our results are consistent with the result of Long et al. 1998 [61].

COL transcription factors were reported to be involved in regulating flowering time in the photoperiod signaling pathway [62]. Besides, the *COL* gene family also plays an important role in a wide range of biological functions, including cell and seedling growth [10, 27, 63], dormancy [64], tuberization [21]. Various functions of this gene family in plant developmental process can be predicted by analyzing their expression profiles. Therefore, we investigated the expression profiles of *COL* genes in different tissues. Differences in expression patterns of *COL* genes in petunia species in different organs/tissues reflected their functional differences (Fig. 4). Except for the *PaCOL5* and *PaCOL10*, the other thirteen genes were expressed in different tissues examined, e.g., leaves, stem bud, root, flower bud, full blooming flower and senescing flower. The average expression levels also varied among different tissues. This observation strongly suggested that the genes preferentially express in any particular tissue may play a critical role in growth and development of that organ in petunia which is a subject of further functional investigation. Notably, *PaCOL5* and *PaCOL10* genes had lower level of expression in the selected samples in this study but differential expression was observed under different stress treatments. Such as *PaCOL10* was induced by heat (41 °C), cold, drought, waterlogging and salinity stress and *PaCOL5* was slightly changed by waterlogging and salinity stress treatment at different time point. These result indicated the spatiotemporal expression of these genes which may express in other developmental stage in different environmental condition [65].

*Stem* is the structural axes of plant which provides the architecture of the above-ground plant parts and thereby assists plant growth under normal and adverse conditions [66]. Preferential expression of *PaCOL3, PaCOL7, PaCOL12, PaCOL14, PaCOL15* genes in stem bud implies that these genes are involved in the development of stem (Fig. 4). Similarly, genes those are highly expressed in leaf and shoot buds could be important for shoot development. Formation of the floral buds is a bridging stage between the vegetative to reproductive phases [67]. A higher transcript abundance of *PaCOL1*, *PaCOL2*, *PaCOL12* and *PaCOL15* in flower buds and that in *PaCOL7*, *PaCOL8, PaCOL13, PaCOL14*, *PaCOL15* in flower buds, full blooming flowers and senescing flowers indicated their possible functions in floral bud formation and flower development (Fig. 4). Functional divergence of the *COL* family genes has been reported in *Arabidopsis,* for example, *AtCOL3* gene influences root growth and lateral root formation but suppresses flowering in *Arabidopsis thaliana* [27]. Altered expressions of *AtCOL1*, *AtCOL2* and *AtCOL9* genes in *Arabidopsis thaliana* accelerated the circadian clock and overexpression of *AtCOL9* gene repressed flowering through the repression of Arabidopsis CONSTANS (AtCO) [23, 30]. Ghd2 (grain number, plant height, and heading date2), a *CO-like* gene increases the yield potential under normal growth condition in rice [55]. Therefore, diverse expression patterns of the *PaCOL* genes strongly suggested diverse functional roles of *PaCOL*s in multiple aspects of growth and development. Moreover, the putative functional analysis found that three genes *PaCOL4, PaCOL8* and *PaCOL13* are involved in the regulation of flower development in petunia (Table S3).

Plant growth and productivity are constantly threatened by various abiotic factors like heat, cold, drought, salinity and ABA. *CO-like* genes were previously studied intensively focusing their potential roles in photoperiodic flowering, but the functionality of those genes in relation to abiotic stresses has not been studied intensively. However, several *COL* genes were reported to be involved in stress response of plant besides regulating flowering times and plant development [33, 55, 68]. For example, Ghd7 (grain number, plant height, and heading date7), a homologue of Ghd2 (a *CO-like* gene), reported to regulate stress tolerance other than controlling plant height, heading date and grain number in rice [55, 68]. In *Arabidopsis*, AtCOL4 improves salt tolerance [33]. In support of previous findings the transcript levels of 15 *PaCOL* genes were investigated under different abiotic and phytohormone stresses. The expression levels of nine *PaCOL* genes, including *PaCOL1, PaCOL2, PaCOL7, PaCOL8, PaCOL10, PaCOL11, PaCOL13, PaCOL14, PaCOL15* altered sharply under different stresses indicating their involvement in stress responsiveness (Figs. 5d, 6, 7). Three genes *PaCOL8, PaCOL10, PaCOL13* markedly up-regulated at 9 h after the treatment of heat, cold and drought stresses suggesting that these six genes may play role in petunia tolerance to heat, cold and drought stresses (Fig. 5, b, c). Besides, *PaCOL7, PaCOL8, PaCOL10, PaCOL13, PaCOL15* showed remarkable expression at 12 h, 24 h and 48 h especially at 24 h under waterlogging stress underscoring their potential role in waterlogging stress (Fig. 5d). A remarkable number of *PaCOL* genes were differentially expressed after salinity treatment among those *PaCOL2* sharply elevated under salt treatment at all the time points suggesting the gene as a good candidate of salinity stress tolerance in petunia (Fig. 6).

ABA is an important phytohormone that plays a regulatory role in response to heat, salinity, and drought [69, 70]. Five *PaCOL* genes (*PaCOL3, PaCOL6, PaCOL7, PaCOL8, PaCOL13, PaCOL15)* were sharply induced at different time points under ABA treatment (Fig. 6) suggesting their involvement in the regulation of abiotic stress tolerance through an ABA-dependent pathway [33]. AtCOL4 belonging to phylogenetic group II was up-regulated under salt, ABA, and osmotic stress which corresponded to the response of the *PaCOL13, PaCOL15* indicating that the genes from phylogenetic group II might play significant role in ABA stress response [33].

*cis*-acting regulatory elements involved in transcriptional regulation of the gene activities and regulate the related gene expression by controlling the efficiency of the promoters [71]. The *cis*-regulatory elements correlated with the transcription factors activate the stress tolerance mechanism and thereby provide tolerance to salt stress in tomato [72]. Several phytohormones and stress responsive *cis*-regulatory elements such as, ABRE, CGTCA-motif, P-box, MBS, AuxRR-core, TC-rich repeats, TGA-element, TCA-element, TGACG-motif, LTR etc. were identified in the putative promoter regions of different *PaCOL* genes (Table S2). Moreover, the expression of *PaCOL* genes was likely to be induced under such phytohormone or abiotic stresses (Fig. 7, Table S2). For example, *PaCOL7, PaCOL8* and *PaCOL13* bearing the ABA responsive elements (ABRE) and drought-inducible elements (MBS) were highly up-regulated at 9 h and 24 h after the ABA and drought treatment compared to control (Fig. 7). The ABA responsive elements (ABRE) are involved in the expression of ABA-dependent genes and thus they are associated with plant adaptation to drought [73]. Taken together, these results indicated that there is a possible correlation for the presence of *cis*-acting elements and gene expression pattern of *PaCOL* genes that may respond to drought stress either through ABA dependent or independent pathways [74].

## Conclusion

In this study, 15 *COL* genes were identified from *P. axillaris* and 18 from *P. inflata.* Subsequently the phylogenetic relationship, gene structure, and organ specific expression analysis of *P. axillaris* indicated that the *COL* gene family might have diverse functions in various aspects of plant growth, stress response and flower bud development besides controlling the flowering time. Moreover, the study indicated a possible relation between the stress responsive *cis*-acting regulatory elements and expression pattern of *PaCOL* genes which laid a foundation for further research on the function of *PaCOL* genes in response to related abiotic stresses.

## Supplementary Information

**Additional file 1: Table S1.** List of primer used in the study. **Table S2.** The cis-regulatory element analysis on the promoter regions of *PaCOL* genes. **Table S3.** Functional analysis of *PaCOL* genes on the basis of Gene Ontology (GO) terms assigned to various genes using BLAST2GO tool. GO terms enrichments in 3 different categories i.e. i) Cellular Component, ii) Molecular Function and iii) Biological Process were predicted. **Table S4.** Ks, Ka, and Ka/Ks ratios calculated for paralogous pairs of petunia *COL* genes. **Table S5.** Sequence analysis of petunia COL proteins identified in *Petunia infrata* genome. **Table S6.** Parameter and program versions used in bioinformatics analysis.

**Additional file 2: Table S7.** COL genes identified in representative plants.

**Additional file 3: Figure S1.** Sequence alignment of COL proteins from petunia, *Arabidopsis* and rice according to the presence of domains. Each letter represents one amino acid, and the left column corresponds to the name of the gene. The BBOX1 and BBOX2 domains are indicated by the blue and green line and CCT domains are indicated by the red line respectively. The red region indicates residues conserved only in the BBOX1 domain, the green region indicates residues conserved in the BBOX2 domain and the indigo region indicates residues conserved in the CCT domain of CO-like proteins. **Figure S2.** Domain architecture of PaCOL proteins. BBOX and CCT domains are indicated by mint and purple color respectively. The black middle region represents the diverge region of COL proteins. **Figure S3.** Analysis of motif composition in PaCOL proteins. Different motifs were represented by different color boxes. MEME database was used for motif analysis with the complete amino acid sequences of PaCOL proteins. **Figure S4.** Phylogenetic analysis of the COL proteins of *Petunia inflata,* Arabidosis, rice, maize, tomato and *Physcomitrella patens*. The phylogenetic tree was established with entire protein sequences from the above plant species by MEGA 7.0 software using the Maximum likelihood method following the complete deletion procedure. The numbers on the branches indicate bootstrap support values from 1000 replications. The scale represents the units of the number of amino acid substitutions per site. **Figure S5.** Analysis of gene structure in *PiCOL*. Blue boxes, black boxes, and red lines indicate exons, upstream/downstream, and introns, respectively. **Figure S6.** Electrophoresis gel image of different organ samples.

## Data Availability

We declare that the dataset(s) supporting the conclusions of this article are included within the article and its additional file(s) will be available in journal web page. The petunia protein sequences used in this study was obtained from solgenomics (https://solgenomics.net/). Protein sequences of COL of *Arabidopsis* and rice were downloaded from TAIR (http://www.arabidopsis.org/) and TIGR (http://rice.plantbiology.msu.edu/) databases respectively. Protein sequences of maize, tomato, and *Physcomitrium patens* were acquired from the NCBI (https://www.ncbi.nlm.nih.gov/) and Sol Genomics databases (https://www.sgn.cornell.edu/) and Phytozome database (https://phytozome.jgi.doe.gov/pz/portal.html) respectively. The phylogenetic data in our manuscript has been deposited into **Dryad repository** with the access URL is https://datadryad.org/stash/share/J_OtXSKDIYM5fI0x7POXI6-zsLOYHxYEzqlwImlP4Lk.

## Abbreviations

CO: CONSTANS
BP: Biological process
CP: Cellular component
GO: Gene Ontology
TOC1: Timing of CAB Expression 1
LD: Long day
Hd1: Heading date 1
SD: Short-day
FT: Flowering Locus T
HD3: Heading date 3
MW: Molecular weight
pI: Isoelectric point
ORF: Open reading frame
GRAVY: Grand average of hygropathicity

## References

[1] Steinbach Y (2019). The Arabidopsis thalianaCONSTANS-LIKE 4 (COL4)–a modulator of flowering time. Front Plant Sci.

[2] Li Y, Xu M (2017). CCT family genes in cereal crops: a current overview. The Crop Journal.

[3] Wu F, Price BW, Haider W, Seufferheld G, Nelson R, Hanzawa Y (2014). Functional and evolutionary characterization of the CONSTANS gene family in short-day photoperiodic flowering in soybean. PLoS One.

[4] Yanovsky MJ, Kay SA (2002). Molecular basis of seasonal time measurement in Arabidopsis. Nature.

[5] Robson F, Costa MMR, Hepworth SR, Vizir I, Pineiro M, Reeves PH, Putterill J, Coupland G (2001). Functional importance of conserved domains in the flowering-time gene CONSTANS demonstrated by analysis of mutant alleles and transgenic plants. Plant J.

[6] Griffiths S, Dunford RP, Coupland G, Laurie DA (2003). The evolution of CONSTANS-like gene families in barley, rice, and Arabidopsis. Plant Physiol.

[7] Nemoto Y, Kisaka M, Fuse T, Yano M, Ogihara Y (2003). Characterization and functional analysis of three wheat genes with homology to the CONSTANS flowering time gene in transgenic rice. Plant J.

[8] Fu J, Yang L, Dai S (2015). Identification and characterization of the CONSTANS-like gene family in the short-day plant Chrysanthemum lavandulifolium. Mol Gen Genomics.

[9] Song X, Duan W, Huang Z, Liu G, Wu P, Liu T, Li Y, Hou X (2015). Comprehensive analysis of the flowering genes in Chinese cabbage and examination of evolutionary pattern of CO-like genes in plant kingdom. Sci Rep.

[10] Liu T, Zhu S, Tang Q, Tang S (2015). Identification of a CONSTANS homologous gene with distinct diurnal expression patterns in varied photoperiods in ramie (Boehmeria nivea L. gaud). Gene.

[11] Gangappa SN, Botto JF (2014). The BBX family of plant transcription factors. Trends Plant Sci.

[12] Koornneef M, Hanhart C, Van der Veen J (1991). A genetic and physiological analysis of late flowering mutants in Arabidopsis thaliana. Mol Gen Genet MGG.

[13] Sibout R, Plantegenet S, Hardtke CS (2008). Flowering as a condition for xylem expansion in Arabidopsis hypocotyl and root. Curr Biol.

[14] Ando E, Ohnishi M, Wang Y, Matsushita T, Watanabe A, Hayashi Y, Fujii M, Ma JF (2013). Inoue S-i, Kinoshita T: TWIN SISTER OF FT, GIGANTEA, and CONSTANS have a positive but indirect effect on blue light-induced stomatal opening in Arabidopsis. Plant Physiol.

[15] Wong A, Hecht VF, Picard K, Diwadkar P, Laurie RE, Wen J, Mysore K, Macknight RC, Weller JL (2014). Isolation and functional analysis of CONSTANS-LIKE genes suggests that a central role for CONSTANS in flowering time control is not evolutionarily conserved in Medicago truncatula. Front Plant Sci.

[16] Izawa T, Oikawa T, Sugiyama N, Tanisaka T, Yano M, Shimamoto K (2002). Phytochrome mediates the external light signal to repress FT orthologs in photoperiodic flowering of rice. Genes Dev.

[17] Kojima S, Takahashi Y, Kobayashi Y, Monna L, Sasaki T, Araki T, Yano M (2002). Hd3a, a rice ortholog of the Arabidopsis FT gene, promotes transition to flowering downstream of Hd1 under short-day conditions. Plant Cell Physiol.

[18] Yamaguchi A, Kobayashi Y, Goto K, Abe M, Araki T (2005). TWIN SISTER OF FT (TSF) acts as a floral pathway integrator redundantly with FT. Plant Cell Physiol.

[19] Sawa M, Nusinow DA, Kay SA, Imaizumi T (2007). FKF1 and GIGANTEA complex formation is required for day-length measurement in Arabidopsis. Science.

[20] Hayama R, Coupland G (2004). The molecular basis of diversity in the photoperiodic flowering responses of Arabidopsis and rice. Plant Physiol.

[21] Böhlenius H, Huang T, Charbonnel-Campaa L, Brunner AM, Jansson S, Strauss SH, Nilsson O (2006). CO/FT regulatory module controls timing of flowering and seasonal growth cessation in trees. Science.

[22] Ballerini ES, Kramer EM (2011). In the light of evolution: a reevaluation of conservation in the CO–FT regulon and its role in photoperiodic regulation of flowering time. Front Plant Sci.

[23] Ledger S, Strayer C, Ashton F, Kay SA, Putterill J (2001). Analysis of the function of two circadian-regulated CONSTANS-LIKE genes. Plant J.

[24] Almada R, Cabrera N, Casaretto JA, Ruiz-Lara S, Villanueva EG (2009). VvCO and VvCOL1, two CONSTANS homologous genes, are regulated during flower induction and dormancy in grapevine buds. Plant Cell Rep.

[25] Takase T, Kakikubo Y, Nakasone A, Nishiyama Y, Yasuhara M, Tokioka-Ono Y, Kiyosue T (2011). Characterization and transgenic study of CONSTANS-LIKE8 (COL8) gene in Arabidopsis thaliana: expression of 35S: COL8 delays flowering under long-day conditions. Plant Biotechnology.

[26] Zhang J-X, Wu K-L, Tian L-N, Zeng S-J, Duan J (2011). Cloning and characterization of a novel CONSTANS-like gene from Phalaenopsis hybrida. Acta Physiol Plant.

[27] Datta S, Hettiarachchi G, Deng X-W, Holm M (2006). Arabidopsis CONSTANS-LIKE3 is a positive regulator of red light signaling and root growth. Plant Cell.

[28] Hassidim M, Harir Y, Yakir E, Kron I, Green RM (2009). Over-expression of CONSTANS-LIKE 5 can induce flowering in short-day grown Arabidopsis. Planta.

[29] Ingkasuwan P, Netrphan S, Prasitwattanaseree S, Tanticharoen M, Bhumiratana S, Meechai A, Chaijaruwanich J, Takahashi H, Cheevadhanarak S (2012). Inferring transcriptional gene regulation network of starch metabolism in Arabidopsis thaliana leaves using graphical Gaussian model. BMC Syst Biol.

[30] Cheng XF, Wang ZY (2005). Overexpression of COL9, a CONSTANS-LIKE gene, delays flowering by reducing expression of CO and FT in Arabidopsis thaliana. Plant J.

[31] González-Schain ND, Díaz-Mendoza M, Żurczak M, Suárez-López P (2012). Potato CONSTANS is involved in photoperiodic tuberization in a graft-transmissible manner. Plant J.

[32] Chen J, Chen JY, Wang JN, Kuang JF, Shan W, Lu WJ (2012). Molecular characterization and expression profiles of MaCOL1, a CONSTANS-like gene in banana fruit. Gene.

[33] Min JH, Chung JS, Lee KH, Kim CS (2015). The CONSTANS-like 4 transcription factor, AtCOL4, positively regulates abiotic stress tolerance through an abscisic acid-dependent manner in Arabidopsis. J Integr Plant Biol.

[34] Cao Z, Guo Y, Yang Q, He Y, Fetouh MI, Warner RM, Deng Z (2019). Genome-wide identification of quantitative trait loci for important plant and flower traits in petunia using a high-density linkage map and an interspecific recombinant inbred population derived from Petunia integrifolia and P. axillaris. Horticulture Res.

[35] Zenoni S, D’Agostino N, Tornielli GB, Quattrocchio F, Chiusano ML, Koes R, Zethof J, Guzzo F, Delledonne M, Frusciante L (2011). Revealing impaired pathways in the an11 mutant by high-throughput characterization of Petunia axillaris and *Petunia inflata* transcriptomes. Plant J.

[36] Bombarely A, Moser M, Amrad A, Bao M, Bapaume L, Barry C (2016). Insight into the evolution of the Solanaceae from the parental genomes of Petunia hybrida. Nat Plants.

[37] Adams S, Hadley P, Pearson S (1998). The effects of temperature, photoperiod, and photosynthetic photon flux on the time to flowering of PetuniaExpress blush pink. J Am Soc Hortic Sci.

[38] Plringer A, Cathey H (1960). Effect of photoperiod, kind of supplemental light and temperature on the growth and flowering of petunia plants. Proc Am Soc Horticultural Sci.

[39] Erwin J. Factors affecting flowering in ornamental plants. In: Flower Breeding and Genetics. Springer; 2007:7–48. 10.1007/978-1-4020-4428-1_1.

[40] Kong X, Lv W, Jiang S, Zhang D, Cai G, Pan J, Li D (2013). Genome-wide identification and expression analysis of calcium-dependent protein kinase in maize. BMC Genomics.

[41] Wang L, Guo K, Li Y, Tu Y, Hu H, Wang B, Cui X, Peng L (2010). Expression profiling and integrative analysis of the CESA/CSL superfamily in rice. BMC Plant Biol.

[42] Kumar S, Stecher G, Tamura K (2016). MEGA7: molecular evolutionary genetics analysis version 7.0 for bigger datasets. Mol Biol Evol.

[43] Lescot M, Déhais P, Thijs G, Marchal K, Moreau Y, Van de Peer Y, Rouzé P, Rombauts S (2002). PlantCARE, a database of plant cis-acting regulatory elements and a portal to tools for in silico analysis of promoter sequences. Nucleic Acids Res.

[44] Conesa A, Götz S (2008). Blast2GO: a comprehensive suite for functional analysis in plant genomics. Int J Plant Genomics.

[45] Cnudde F, Moretti C, Porceddu A, Pezzotti M, Gerats T (2003). Transcript profiling on developing Petunia hybrida floral organs. Sex Plant Reprod.

[46] Ke Y-T, Lin K-F, Gu C-H, Yeh C-H (2020). Molecular characterization and expression profile of PaCOL1, a CONSTANS-like gene in Phalaenopsis orchid. Plants.

[47] Aoki K, Yano K, Suzuki A, Kawamura S, Sakurai N, Suda K, Kurabayashi A, Suzuki T, Tsugane T, Watanabe M (2010). Large-scale analysis of full-length cDNAs from the tomato (Solanum lycopersicum) cultivar Micro-tom, a reference system for the Solanaceae genomics. BMC Genomics.

[48] Schmittgen TD, Livak KJ (2008). Analyzing real-time PCR data by the comparative C T method. Nat Protoc.

[49] Khatun K, Nath UK, Park J-I, Kim CK, Nou IS, Chung M-Y (2018). Expression profiling of the CSDP transcription factor gene family points to roles in organ development and abiotic stress response in tomato (Solanum lycopersicum L.). Plant Mol Biol Report.

[50] Hu T, Wei Q, Wang W, Hu H, Mao W, Zhu Q, Bao C (2018). Genome-wide identification and characterization of CONSTANS-like gene family in radish (Raphanus sativus). PLoS One.

[51] Nei M, Gojobori T (1986). Simple methods for estimating the numbers of synonymous and nonsynonymous nucleotide substitutions. Mol Biol Evol.

[52] Koch MA, Haubold B, Mitchell-Olds T (2000). Comparative evolutionary analysis of chalcone synthase and alcohol dehydrogenase loci in Arabidopsis, Arabis, and related genera (Brassicaceae). Mol Biol Evol.

[53] Nakashima K, Ito Y, Yamaguchi-Shinozaki K (2009). Transcriptional regulatory networks in response to abiotic stresses in Arabidopsis and grasses. Plant Physiol.

[54] Zhang Z, Yu J (2006). Evaluation of six methods for estimating synonymous and nonsynonymous substitution rates. Genomics Proteomics Bioinformatics.

[55] Liu J, Shen J, Xu Y, Li X, Xiao J, Xiong L (2016). Ghd2, a CONSTANS-like gene, confers drought sensitivity through regulation of senescence in rice. J Exp Bot.

[56] Schnable PS, Ware D, Fulton RS, Stein JC, Wei F, Pasternak S, Liang C, Zhang J, Fulton L, Graves TA (2009). The B73 maize genome: complexity, diversity, and dynamics. Science.

[57] Zhao Y, Zhou Y, Jiang H, Li X, Gan D, Peng X, Zhu S, Cheng B (2011). Systematic analysis of sequences and expression patterns of drought-responsive members of the HD-zip gene family in maize. PLoS One.

[58] Quraishi UM, Abrouk M, Murat F, Pont C, Foucrier S, Desmaizieres G, Confolent C, Riviere N, Charmet G, Paux E (2011). Cross-genome map based dissection of a nitrogen use efficiency ortho-metaQTL in bread wheat unravels concerted cereal genome evolution. Plant J.

[59] Qin W, Yu Y, Jin Y, Wang X, Liu J, Xi J, Li Z, Li H, Zhao G, Hu W (2018). Genome-wide analysis elucidates the role of CONSTANS-like genes in stress responses of cotton. Int J Mol Sci.

[60] Nguyen HD, Yoshihama M, Kenmochi N (2006). Phase distribution of spliceosomal introns: implications for intron origin. BMC Evol Biol.

[61] Long M, De Souza SJ, Rosenberg C, Gilbert W (1998). Relationship between “proto-splice sites” and intron phases: evidence from dicodon analysis. Proc Natl Acad Sci.

[62] Shimizu M, Ichikawa K, Aoki S (2004). Photoperiod-regulated expression of the PpCOL1 gene encoding a homolog of CO/COL proteins in the moss Physcomitrella patens. Biochem Biophys Res Commun.

[63] Datta S, Hettiarachchi C, Johansson H, Holm M (2007). SALT TOLERANCE HOMOLOG2, a B-box protein in Arabidopsis that activates transcription and positively regulates light-mediated development. Plant Cell.

[64] González-Schain N, Suárez-López P (2008). CONSTANS delays flowering and affects tuber yield in potato. Biol Plant.

[65] Zhang J, Wu J, Guo M, Aslam M, Wang Q, Ma H, Li S, Zhang X, Cao S (2020). Genome-wide characterization and expression profiling of Eucalyptus grandis HD-zip gene family in response to salt and temperature stress. BMC Plant Biol.

[66] Speck T, Burgert I (2011). Plant stems: functional design and mechanics. Annu Rev Mater Res.

[67] Koutinas N, Pepelyankov G, Lichev V (2010). Flower induction and flower bud development in apple and sweet cherry. Biotechnology Biotechnological Equipment.

[68] Xue W, Xing Y, Weng X, Zhao Y, Tang W, Wang L, Zhou H, Yu S, Xu C, Li X (2008). Natural variation in Ghd7 is an important regulator of heading date and yield potential in rice. Nat Genet.

[69] Huang Y-C, Niu C-Y, Yang C-R, Jinn T-L (2016). The heat stress factor HSFA6b connects ABA signaling and ABA-mediated heat responses. Plant Physiol.

[70] Suzuki N, Bassil E, Hamilton JS, Inupakutika MA, Zandalinas SI, Tripathy D, Luo Y, Dion E, Fukui G, Kumazaki A (2016). ABA is required for plant acclimation to a combination of salt and heat stress. PLoS One.

[71] Lu Z, Ricci WA, Schmitz RJ, Zhang X (2018). Identification of cis-regulatory elements by chromatin structure. Curr Opin Plant Biol.

[72] Yang R, Liu J, Lin Z, Sun W, Wu Z, Hu H, Zhang Y (2018). ERF transcription factors involved in salt response in tomato. Plant Growth Regul.

[73] Hubbard KE, Siegel RS, Valerio G, Brandt B, Schroeder JI (2012). Abscisic acid and CO2 signalling via calcium sensitivity priming in guard cells, new CDPK mutant phenotypes and a method for improved resolution of stomatal stimulus–response analyses. Ann Bot.

[74] Cao L, Zhang P, Lu X, Wang G, Wang Z, Zhang Q, Zhang X, Wei X, Mei F, Wei L (2020). Systematic Analysis of the Maize OSCA Genes Revealing ZmOSCA Family Members Involved in Osmotic Stress and ZmOSCA2. 4 Confers Enhanced Drought Tolerance in Transgenic Arabidopsis. Int J Mol Sci.

