# Supplementary material for: Genome-wide identification, genomic organization, and expression profiling of the CONSTANS-like (COL) gene family in petunia under multiple stresses
Source: BMC Genomics. 2021 Oct 8;22:727. doi: 10.1186/s12864-021-08019-w (PMC8499527; doi:10.1186/s12864-021-08019-w)
Supplement: Supplementary file 1 — Additional file 1: Table S1. List of primer used in the study. Table S2. The cis-regulatory element analysis on the promoter regions of PaCOL genes. Table S3. Functional analysis of PaCOL genes on the basis of Gene Ontology (GO) terms assigned to various genes using BLAST2GO tool. GO terms enrichments in 3 different categories i.e. i) Cellular Component, ii) Molecular Function and iii) Biological Process were predicted. Table S4. Ks, Ka, and Ka/Ks ratios calculated for paralogous pairs of petunia COL genes. Table S5. Sequence analysis of petunia COL proteins identified in Petunia infrata genome. Table S6. Parameter and program versions used in bioinformatics analysis. [file 12864_2021_8019_MOESM1_ESM.docx]

| **Gene Name** | **Forward primer** | **Reverse primer** |
| --- | --- | --- |
| *PaCOL1* | AACTCCCTCTTTGGAGCCTA | ATTAGAACCCTCTGGGAAGT |
| *PaCOL2* | AGCCTCCCTAAGCAACATTG | GACTTTGTTGTGCACTGCCG |
| *PaCOL3* | TTGCCCTGCTGATGATGCCT | GGTTGACAATTTTGGTCGGA |
| *PaCOL4* | ACTACTTGGATCTTGGAGAG | CTGCTGCTGCTGTTGATGTT |
| *PaCOL5* | GTGACTCGTGCAAAACAACG | ACATGTCACGCAAAGAGCAG |
| *PaCOL6* | GAGGTGGACATGAGTATTGA | CACCCCGAGAGCTGGAGTCA |
| *PaCOL7* | GCTGTGTCACGCTACAAGGA | CTACTGATGCATGGGGGAAT |
| *PaCOL8* | AGTGTGACTCAGCAGGTTGA | CTGAGTGTTCTGATCATAGG |
| *PaCOL9* | AGGTGAGCAATTCTGGAACG | CCTTCGTTCTCAAACAGCAG |
| *PaCOL10* | CCCGCTTCATCTTGTCCCCG | CCCGTCTTCGCCTCATTCGT |
| *PaCOL11* | CAGAGATCCAGCACCCTTCT | GGCCAAAAGCTCATAACCAC |
| *PaCOL12* | ATCTCATGGTGGTTCTGAAG | TGTATTCAACCTCTTCTCCA |
| *PaCOL13* | GCCTAATCTCGACCAAAAGC | CGAGGGAACCACTGAATAGC |
| *PaCOL14* | ACACCCTCAACCCCACTACA | AGTGATGGGGTGGTGAAAGA |
| *PaCOL15* | CTCAGCAACATCTTGGCACA | TCTCAGGGACGACAAGATGA |
| *EF1α* | CCTGGTCAAATTGGAAACGG | CAGATCGCCTGTCAATCTTGG |
| Peaxi162Scf00074g02025 | GCACTGGTGACTTGCAGAAA | GGTCGATTGTCGGCTAATGT |
| Peaxi162Scf00420g00155 | GCTAGTAATCAGCAGCTCAA | AGCCGTCCGTACTAGCTCAC |

**Table S1** List of primer used in the study

**Table S2** The cis-regulatory element analysis on the promoter regions of *PaCOL* genes

| **Gene name** | **Cis element** | **Related organism** | **Position** | **Strand** | **Matrix score** | **Sequence** | **Function** |
| --- | --- | --- | --- | --- | --- | --- | --- |
| ***PaCOL1*** | **A BRE** | *Arabidopsis thaliana* | 700 | + | 6 | CACGTG | cis-acting element involved in the abscisic acid responsiveness |
|  | AE-box | *Arabidopsis thaliana* | 61 | + | 8 | AGAAACAA | part of a module for light response |
|  | ARE | *Zea mays* | 150 | - | 6 | AAACCA | cis-acting regulatory element essential for the anaerobic induction |
|  | ATCT-motif | *Pisum sativum* | 1078 | - | 9 | AATCTAATCC | part of a conserved DNA module involved in light responsiveness |
|  | Box 4 | *Petroselinum crispum* | 884 | - | 6 | ATTAAT | part of a conserved DNA module involved in light responsiveness |
|  | CAAT-box | *Arabidopsis thaliana* | 41 | + | 5 | CCAAT | common cis-acting element in promoter and enhancer regions |
|  | CGTCA-motif | *Hordeum vulgare* | 178 | - | 5 | CGTCA | cis-acting regulatory element involved in the MeJA-responsiveness |
|  | G-Box | *Pisum sativum* | 700 | + | 6 | CACGTG | cis-acting regulatory element involved in light responsiveness |
|  | G-box | *Arabidopsis thaliana* | 698 | - | 9 | GCCACGTGGA | cis-acting regulatory element involved in light responsiveness |
|  | GC-motif | *Zea mays* | 18 | + | 6 | CCCCCG | enhancer-like element involved in anoxic specific inducibility |
|  | GT1-motif | *vena sativa* | 916 | + | 7 | GGTTAAT | light responsive element |
|  | LAMP-element | *Pisum sativum* | 1421 | - | 8 | CTTTATCA | part of a light responsive element |
|  | MBS | *Arabidopsis thaliana* | 138 | - | 6 | CAACTG | MYB binding site involved in drought-inducibility |
|  | MBSI | *Petunia hybrida* | 361 | + | 10.5 | aaaAaaC(G/C)GTTA | MYB binding site involved in flavonoid biosynthetic genes regulation |
|  | P-box | *Oryza sativa* | 66 | - | 7 | CCTTTTG | gibberellin-responsive element |
|  | TATA-box | *Oryza sativa* | 325 | - | 7 | TACAAAA | core promoter element around -30 of transcription start |
|  | TATC-box | *Oryza sativa* | 630 | - | 7 | TATCCCA | cis-acting element involved in gibberellin-responsiveness |
|  | TCT-motif | *Arabidopsis thaliana* | 1322 | + | 6 | TCTTAC | part of a light responsive element |
|  | circadian | *Lycopersicon esculentum* | 633 | - | 9 | CAAAGATATC | cis-acting regulatory element involved in circadian control |
| ***PaCOL2*** | ABRE | *Arabidopsis thaliana* | 457 | + | 6 | CACGTG | cis-acting element involved in the abscisic acid responsiveness |
|  | ARE | *Zea mays* | 566 | - | 6 | AAACCA | cis-acting regulatory element essential for the anaerobic induction |
|  | AuxRR-core | *Nicotiana tabacum* | 1283 | - | 7 | GGTCCAT | cis-acting regulatory element involved in auxin responsiveness |
|  | Box 4 | *Petroselinum crispum* | 740 | + | 6 | ATTAAT | part of a conserved DNA module involved in light responsiveness |
|  | CAAT-box | *Arabidopsis thaliana* | 126 | + | 10 | CAACCAACTCC | common cis-acting element in promoter and enhancer regions |
|  | CAT-box | *Arabidopsis thaliana* | 628 | + | 6 | GCCACT | cis-acting regulatory element related to meristem expression |
|  | CGTCA-motif | *Hordeum vulgare* | 706 | + | 5 | CGTCA | cis-acting regulatory element involved in the MeJA-responsiveness |
|  | G-Box | *Pisum sativum* | 428 | + | 6 | CACGTG | cis-acting regulatory element involved in light responsiveness |
|  | G-box | *Arabidopsis thaliana* | 428 | + | 6 | CACGTG | cis-acting regulatory element involved in light responsiveness |
|  | GA-motif | *Arabidopsis thaliana* | 925 | + | 8 | ATAGATAA | part of a light responsive element |
|  | GATA-motif | *Arabidopsis thaliana* | 208 | - | 7 | GATAGGA | part of a light responsive element |
|  | GT1-motif | *Avena sativa* | 250 | - | 7 | GGTTAAT | light responsive element |
|  | MBS | *Arabidopsis thaliana* | 214 | + | 6 | CAACTG | MYB binding site involved in drought-inducibility |
|  | MRE | *Petroselinum crispum* | 1129 | - | 7 | AACCTAA | MYB binding site involved in light responsiveness |
|  | P-box | *Oryza sativa* | 719 | + | 7 | CCTTTTG | gibberellin-responsive element |
|  | TATA-box | *Brassica oleracea* | 182 | + | 6 | ATATAA | core promoter element around -30 of transcription start |
|  | TGACG-motif | *Hordeum vulgare* | 706 | - | 5 | TGACG | cis-acting regulatory element involved in the MeJA-responsiveness |
|  | circadian | *Lycopersicon esculentum* | 177 | + | 9 | CAAAGATATC | cis-acting regulatory element involved in circadian control |
| ***PaCOL3*** | AAAC-motif | *Spinacia oleracea* | 1078 | + | 11 | CAATCAAAACCT | light responsive element |
|  | ABRE | *Arabidopsis thaliana* | 966 | - | 5 | ACGTG | cis-acting element involved in the abscisic acid responsiveness |
|  | AE-box | *Arabidopsis thaliana* | 1393 | - | 8 | AGAAACAA | part of a module for light response |
|  | ARE | *Zea mays* | 455 | + | 6 | AAACCA | cis-acting regulatory element essential for the anaerobic induction |
|  | CAAT-box | *Arabidopsis thaliana* | 488 | + | 5 | CCAAT | common cis-acting element in promoter and enhancer regions |
|  | CGTCA-motif | *Hordeum vulgare* | 1 | + | 5 | CGTCA | cis-acting regulatory element involved in the MeJA-responsiveness |
|  | G-Box | *Pisum sativum* | 966 | + | 6 | CACGTT | cis-acting regulatory element involved in light responsiveness |
|  | GARE-motif | *Brassica oleracea* | 1468 | + | 7 | TCTGTTG | gibberellin-responsive element |
|  | GATA-motif | *Arabidopsis thaliana* | 1123 | - | 10 | AAGATAAGATT | part of a light responsive element |
|  | GT1-motif | *Arabidopsis thaliana* | 1073 | - | 6 | GGTTAA | light responsive element |
|  | MBS | *Arabidopsis thaliana* | 1241 | + | 6 | CAACTG | MYB binding site involved in drought-inducibility |
|  | TATA-box | *Arabidopsis thaliana* | 1272 | - | 7 | TATATAA | core promoter element around -30 of transcription start |
|  | TCCC-motif | *Spinacia oleracea* | 836 | - | 7 | TCTCCCT | part of a light responsive element |
|  | TCT-motif | *Arabidopsis thaliana* | 608 | + | 6 | TCTTAC | part of a light responsive element |
|  | TGA-element | *Brassica oleracea* | 332 | + | 6 | AACGAC | auxin-responsive element |
|  | TGACG-motif | *Hordeum vulgare* | 1 | - | 5 | TGACG | cis-acting regulatory element involved in the MeJA-responsiveness |
| ***PaCOL4*** | ABRE | *Arabidopsis thaliana* | 1345 | - | 6 | CACGTG | cis-acting element involved in the abscisic acid responsiveness |
|  | ARE | *Zea mays* | 820 | + | 6 | AAACCA | cis-acting regulatory element essential for the anaerobic induction |
|  | Box 4 | *Petroselinum crispum* | 166 | + | 6 | ATTAAT | part of a conserved DNA module involved in light responsiveness |
|  | CAAT-box | *Arabidopsis thaliana* | 56 | - | 5 | CCAAT | common cis-acting element in promoter and enhancer regions |
|  | CAT-box | *Arabidopsis thaliana* | 797 | - | 6 | GCCACT | cis-acting regulatory element related to meristem expression |
|  | G-Box | *Pisum sativum* | 1345 | - | 6 | CACGTG | cis-acting regulatory element involved in light responsiveness |
|  | G-box | *Lycopersicon esculentum* | 1342 | - | 12 | tgACACGTGGCA | cis-acting regulatory element involved in light responsiveness |
|  | GCN4_motif | *Oryza sativa* | 671 | + | 7 | TGAGTCA | cis-regulatory element involved in endosperm expression |
|  | Gap-box | *Arabidopsis thaliana* | 828 | + | 9 | CAAATGAA(A/G)A | part of a light responsive element |
|  | I-box | *Zea mays* | 1317 | - | 9 | gGATAAGGTG | part of a light responsive element |
|  | P-box | *Oryza sativa* | 75 | - | 7 | CCTTTTG | gibberellin-responsive element |
|  | TATA-box | *Brassica napus* | 477 | + | 6 | ATATAT | core promoter element around -30 of transcription start |
|  | TGA-element | *Brassica oleracea* | 914 | - | 6 | AACGAC | auxin-responsive element |
| ***PaCOL5*** | A-box | *Petroselinum crispum* | 1365 | + | 6 | CCGTCC | cis-acting regulatory element |
|  | ABRE | *Arabidopsis thaliana* | 267 | - | 5 | ACGTG | cis-acting element involved in the abscisic acid responsiveness |
|  | ARE | *Zea mays* | 456 | + | 6 | AAACCA | cis-acting regulatory element essential for the anaerobic induction |
|  | ATCT-motif | *Pisum sativum* | 66 | - | 9 | AATCTAATCC | part of a conserved DNA module involved in light responsiveness |
|  | Box 4 | *Petroselinum crispum* | 1294 | - | 6 | ATTAAT | part of a conserved DNA module involved in light responsiveness |
|  | CAAT-box | *Arabidopsis thaliana* | 425 | + | 5 | CCAAT | common cis-acting element in promoter and enhancer regions |
|  | G-box | *Arabidopsis thaliana* | 267 | - | 6 | TACGTG | cis-acting regulatory element involved in light responsiveness |
|  | LAMP-element | *Pisum sativum* | 51 | + | 8 | CTTTATCA | part of a light responsive element |
|  | MBS | *Arabidopsis thaliana* | 1326 | - | 6 | CAACTG | MYB binding site involved in drought-inducibility |
|  | P-box | *Oryza sativa* | 1181 | + | 7 | CCTTTTG | gibberellin-responsive element |
|  | TATA-box | *Arabidopsis thaliana* | 875 | + | 9 | ccTATAAAaa | core promoter element around -30 of transcription start |
|  | TC-rich repeats | *Nicotiana tabacum* | 304 | + | 9 | ATTCTCTAAC | cis-acting element involved in defense and stress responsiveness |
|  | TCT-motif | *Arabidopsis thaliana* | 1106 | - | 6 | TCTTAC | part of a light responsive element |
|  | TGA-element | *Brassica oleracea* | 400 | - | 6 | AACGAC | auxin-responsive element |
|  | circadian | *Lycopersicon esculentum* | 1378 | - | 9 | CAAAGATATC | cis-acting regulatory element involved in circadian control |
| ***PaCOL6*** | ABRE | *Arabidopsis thaliana* | 614 | - | 5 | ACGTG | cis-acting element involved in the abscisic acid responsiveness |
|  | ARE | *Zea mays* | 484 | + | 6 | AAACCA | cis-acting regulatory element essential for the anaerobic induction |
|  | CAAT-box | *Pisum sativum* | 474 | + | 5 | CAAAT | common cis-acting element in promoter and enhancer regions |
|  | CAG-motif | *Arabidopsis thaliana* | 1247 | - | 10 | GAAAGGCAGAC | part of a light response element |
|  | CGTCA-motif | *Hordeum vulgare* | 1346 | - | 5 | CGTCA | cis-acting regulatory element involved in the MeJA-responsiveness |
|  | G-box | *Brassica napus* | 613 | + | 8 | CCACGTAA | cis-acting regulatory element involved in light responsiveness |
|  | I-box | *Solanum tuberosum* | 102 | - | 9 | TGATAATGT | part of a light responsive element |
|  | MBS | *Arabidopsis thaliana* | 58 | + | 6 | CAACTG | MYB binding site involved in drought-inducibility |
|  | TATA-box | *Arabidopsis thaliana* | 266 | - | 8 | TATTTAAA | core promoter element around -30 of transcription start |
|  | TC-rich repeats | *Nicotiana tabacum* | 781 | + | 9 | ATTCTCTAAC | cis-acting element involved in defense and stress responsiveness |
|  | TCA-element | *Nicotiana tabacum* | 315 | - | 9 | CCATCTTTTT | cis-acting element involved in salicylic acid responsiveness |
|  | TCT-motif | *Arabidopsis thaliana* | 1463 | + | 6 | TCTTAC | part of a light responsive element |
|  | TGACG-motif | *Hordeum vulgare* | 1346 | + | 5 | TGACG | cis-acting regulatory element involved in the MeJA-responsiveness |
| ***PaCOL7*** | ABRE | *Arabidopsis thaliana* | 264 | + | 5 | ACGTG | cis-acting element involved in the abscisic acid responsiveness |
|  | ARE | *Zea mays* | 518 | - | 6 | AAACCA | cis-acting regulatory element essential for the anaerobic induction |
|  | AT-rich sequence | *Pisum sativum* | 982 | + | 9 | TAAAATACT | element for maximal elicitor-mediated activation (2copies) |
|  | Box 4 | *Petroselinum crispum* | 215 | + | 6 | ATTAAT | part of a conserved DNA module involved in light responsiveness |
|  | CAAT-box | *Arabidopsis thaliana* | 1187 | - | 5 | CCAAT | common cis-acting element in promoter and enhancer regions |
|  | CAG-motif | *Arabidopsis thaliana* | 642 | + | 10 | GAAAGGCAGAC | part of a light response element |
|  | CGTCA-motif | *Hordeum vulgare* | 790 | - | 5 | CGTCA | cis-acting regulatory element involved in the MeJA-responsiveness |
|  | G-box | *Arabidopsis thaliana* | 263 | + | 6 | TACGTG | cis-acting regulatory element involved in light responsiveness |
|  | I-box | *Flaveria trinervia* | 114 | - | 10 | cCATATCCAAT | part of a light responsive element |
|  | TATA-box | *Brassica napus* | 40 | + | 6 | ATATAT | core promoter element around -30 of transcription start |
|  | TC-rich repeats | *Nicotiana tabacum* | 1307 | + | 9 | ATTCTCTAAC | cis-acting element involved in defense and stress responsiveness |
|  | TCT-motif | *Arabidopsis thaliana* | 82 | - | 6 | TCTTAC | part of a light responsive element |
|  | TGA-element | *Brassica oleracea* | 1407 | - | 6 | AACGAC | auxin-responsive element |
|  | TGACG-motif | *Hordeum vulgare* | 790 | + | 5 | TGACG | cis-acting regulatory element involved in the MeJA-responsiveness |
| ***PaCOL8*** | ABRE | *Arabidopsis thaliana* | 997 | + | 5 | ACGTG | cis-acting element involved in the abscisic acid responsiveness |
|  | ARE | *Zea mays* | 88 | - | 6 | AAACCA | cis-acting regulatory element essential for the anaerobic induction |
|  | AT-rich element | *Glycine max* | 258 | - | 10 | ATAGAAATCAA | binding site of AT-rich DNA binding protein (ATBP-1) |
|  | AT-rich sequence | *Pisum sativum* | 898 | + | 9 | TAAAATACT | element for maximal elicitor-mediated activation (2copies) |
|  | CAAT-box | *Pisum sativum* | 980 | + | 5 | CAAAT | common cis-acting element in promoter and enhancer regions |
|  | CGTCA-motif | *Hordeum vulgare* | 1206 | + | 5 | CGTCA | cis-acting regulatory element involved in the MeJA-responsiveness |
|  | G-Box | *Pisum sativum* | 996 | - | 6 | CACGTT | cis-acting regulatory element involved in light responsiveness |
|  | GA-motif | *Arabidopsis thaliana* | 833 | - | 8 | ATAGATAA | part of a light responsive element |
|  | GARE-motif | *Brassica oleracea* | 1090 | - | 7 | TCTGTTG | gibberellin-responsive element |
|  | GATA-motif | *Solanum tuberosum* | 1409 | - | 9 | AAGGATAAGG | part of a light responsive element |
|  | MBS | *Arabidopsis thaliana* | 1235 | + | 6 | CAACTG | MYB binding site involved in drought-inducibility |
|  | O2-site | *Zea mays* | 1240 | + | 9 | GATGATGTGG | cis-acting regulatory element involved in zein metabolism regulation |
|  | TATA-box | *Arabidopsis thaliana* | 60 | + | 8 | TAAAGATT | core promoter element around -30 of transcription start |
|  | TATC-box | *Oryza sativa* | 190 | - | 7 | TATCCCA | cis-acting element involved in gibberellin-responsiveness |
|  | TC-rich repeats | *Nicotiana tabacum* | 207 | - | 9 | ATTCTCTAAC | cis-acting element involved in defense and stress responsiveness |
|  | TCA-element | *rassica oleracea* | 755 | - | 9 | TCAGAAGAGG | cis-acting element involved in salicylic acid responsiveness |
|  | TCCC-motif | *pinacia oleracea* | 633 | + | 7 | TCTCCCT | part of a light responsive element |
|  | TGACG-motif | *Hordeum vulgare* | 1206 | - | 5 | TGACG | cis-acting regulatory element involved in the MeJA-responsiveness |
|  | circadian | *Lycopersicon esculentum* | 867 | - | 9 | CAAAGATATC | cis-acting regulatory element involved in circadian control |
| ***PaCOL9*** | AE-box | *Arabidopsis thaliana* | 703 | + | 8 | AGAAACAA | part of a module for light response |
|  | ARE | *Zea mays* | 1496 | - | 6 | AAACCA | cis-acting regulatory element essential for the anaerobic induction |
|  | ATCT-motif | *Pisum sativum* | 440 | + | 9 | AATCTAATCC | part of a conserved DNA module involved in light responsiveness |
|  | Box 4 | *Petroselinum crispum* | 1364 | - | 6 | ATTAAT | part of a conserved DNA module involved in light responsiveness |
|  | CAAT-box | *Pisum sativum* | 140 | + | 5 | CAAAT | common cis-acting element in promoter and enhancer regions |
|  | CAT-box | *Arabidopsis thaliana* | 255 | - | 6 | GCCACT | cis-acting regulatory element related to meristem expression |
|  | G-box | *Brassica oleracea* | 73 | - | 9 | TAACACGTAG | cis-acting regulatory element involved in light responsiveness |
|  | GT1-motif | *Avena sativa* | 488 | - | 7 | GGTTAAT | light responsive element |
|  | MRE | *Petroselinum crispum* | 554 | + | 7 | AACCTAA | MYB binding site involved in light responsiveness |
|  | P-box | *Oryza sativa* | 2 | + | 7 | CCTTTTG | gibberellin-responsive element |
|  | TATA-box | *Brassica napus* | 575 | + | 6 | ATATAT | core promoter element around -30 of transcription start |
|  | TCCC-motif | *Spinacia oleracea* | 53 | + | 7 | TCTCCCT | part of a light responsive element |
|  | TCT-motif | *Arabidopsis thaliana* | 1147 | - | 6 | TCTTAC | part of a light responsive element |
|  | TGA-element | *Brassica oleracea* | 1425 | - | 6 | AACGAC | auxin-responsive element |
|  | circadian | *Lycopersicon esculentum* | 157 | + | 9 | CAAAGATATC | cis-acting regulatory element involved in circadian control |
| ***PaCOL10*** | ABRE | *Arabidopsis thaliana* | 1013 | + | 5 | ACGTG | cis-acting element involved in the abscisic acid responsiveness |
|  | AE-box | *Arabidopsis thaliana* | 672 | + | 8 | AGAAACAA | part of a module for light response |
|  | ARE | *Zea mays* | 277 | + | 6 | AAACCA | cis-acting regulatory element essential for the anaerobic induction |
|  | Box 4 | *Petroselinum crispum* | 1099 | - | 6 | ATTAAT | part of a conserved DNA module involved in light responsiveness |
|  | CAAT-box | *Arabidopsis thaliana* | 1112 | + | 5 | CCAAT | common cis-acting element in promoter and enhancer regions |
|  | CGTCA-motif | *Hordeum vulgare* | 286 | + | 5 | CGTCA | cis-acting regulatory element involved in the MeJA-responsiveness |
|  | G-box | *Zea mays* | 1012 | - | 6 | CACGTC | cis-acting regulatory element involved in light responsiveness |
|  | GA-motif | *Arabidopsis thaliana* | 1165 | + | 8 | ATAGATAA | part of a light responsive element |
|  | GARE-motif | *Brassica oleracea* | 238 | + | 7 | TCTGTTG | gibberellin-responsive element |
|  | GC-motif | *Zea mays* | 1075 | - | 6 | CCCCCG | enhancer-like element involved in anoxic specific inducibility |
|  | HD-Zip 1 | *Arabidopsis thaliana* | 1316 | - | 8.5 | CAAT(A/T)ATTG | element involved in differentiation of the palisade mesophyll cells |
|  | I-box | *Triticum aestivum* | 1167 | + | 8 | AGATAAGG | part of a light responsive element |
|  | L-box | *Petroselinum crispum* | 1382 | + | 10 | ATCCCACCTAC | part of a light responsive element |
|  | LTR | *Hordeum vulgare* | 113 | + | 6 | CCGAAA | cis-acting element involved in low-temperature responsiveness |
|  | P-box | *Oryza sativa* | 127 | - | 7 | CCTTTTG | gibberellin-responsive element |
|  | TATA-box | *Oryza sativa* | 1342 | + | 8 | TACATAAA | core promoter element around -30 of transcription start |
|  | TCCC-motif | *Spinacia oleracea* | 1366 | + | 7 | TCTCCCT | part of a light responsive element |
|  | TGA-element | *Brassica oleracea* | 254 | + | 6 | AACGAC | auxin-responsive element |
|  | TGACG-motif | *Hordeum vulgare* | 286 | - | 5 | TGACG | cis-acting regulatory element involved in the MeJA-responsiveness |
| ***PaCOL11*** | ABRE | *Arabidopsis thaliana* | 1082 | - | 6 | CACGTG | cis-acting element involved in the abscisic acid responsiveness |
|  | ARE | *Zea mays* | 842 | - | 6 | AAACCA | cis-acting regulatory element essential for the anaerobic induction |
|  | CAAT-box | *Arabidopsis thaliana* | 144 | + | 5 | CCAAT | common cis-acting element in promoter and enhancer regions |
|  | G-Box | *Pisum sativum* | 1082 | - | 6 | CACGTG | cis-acting regulatory element involved in light responsiveness |
|  | G-box | *Brassica oleracea* | 252 | + | 9 | TAACACGTAG | cis-acting regulatory element involved in light responsiveness |
|  | GATA-motif | *Arabidopsis thaliana* | 1331 | - | 10 | AAGATAAGATT | part of a light responsive element |
|  | O2-site | *Zea mays* | 900 | - | 8 | GATGA(C/T)(A/G)TG(A/G) | cis-acting regulatory element involved in zein metabolism regulation |
|  | P-box | *Oryza sativa* | 794 | + | 7 | CCTTTTG | gibberellin-responsive element |
|  | TATA-box | *Oryza sativa* | 1172 | + | 7 | TACAAAA | core promoter element around -30 of transcription start |
|  | circadian | *Lycopersicon esculentum* | 192 | - | 9 | CAAAGATATC | cis-acting regulatory element involved in circadian control |
| ***PaCOL12*** | ABRE | *Arabidopsis thaliana* | 177 | + | 5 | ACGTG | cis-acting element involved in the abscisic acid responsiveness |
|  | ATCT-motif | *Pisum sativum* | 1277 | + | 9 | AATCTAATCC | part of a conserved DNA module involved in light responsiveness |
|  | Box 4 | *Petroselinum crispum* | 210 | + | 6 | ATTAAT | part of a conserved DNA module involved in light responsivenes |
|  | CAAT-box | *Pisum sativum* | 368 | + | 5 | CAAAT | common cis-acting element in promoter and enhancer regions |
|  | CGTCA-motif | *Hordeum vulgare* | 158 | - | 5 | CGTCA | cis-acting regulatory element involved in the MeJA-responsiveness |
|  | G-Box | *Pisum sativum* | 176 | + | 6 | CACGTG | cis-acting regulatory element involved in light responsiveness |
|  | G-box | *Arabidopsis thaliana* | 176 | + | 6 | CACGTG | cis-acting regulatory element involved in light responsiveness |
|  | GA-motif | *Arabidopsis thaliana* | 836 | + | 8 | ATAGATAA | part of a light responsive element |
|  | MBS | *Arabidopsis thaliana* | 1044 | - | 6 | CAACTG | MYB binding site involved in drought-inducibility |
|  | TATA-box | *Oryza sativa* | 18 | - | 7 | TACAAAA | core promoter element around -30 of transcription start |
|  | TC-rich repeats | *Nicotiana tabacum* | 220 | - | 9 | ATTCTCTAAC | cis-acting element involved in defense and stress responsiveness |
|  | TCT-motif | *Arabidopsis thaliana* | 489 | - | 6 | TCTTAC | part of a light responsive element |
|  | TGA-element | *Brassica oleracea* | 171 | + | 6 | AACGAC | auxin-responsive element |
|  | TGACG-motif | *Hordeum vulgare* | 158 | + | 5 | TGACG | cis-acting regulatory element involved in the MeJA-responsiveness |
|  | circadian | *Lycopersicon esculentum* | 123 | + | 9 | CAAAGATATC | cis-acting regulatory element involved in circadian control |
| ***PaCOL13*** | ABRE | *Arabidopsis thaliana* | 1082 | - | 6 | CACGTG | cis-acting element involved in the abscisic acid responsiveness |
|  | ARE | *Zea mays* | 646 | + | 6 | AAACCA | cis-acting regulatory element essential for the anaerobic induction |
|  | CAAT-box | *Pisum sativum* | 163 | + | 5 | CAAAT | common cis-acting element in promoter and enhancer regions |
|  | CAT-box | *rabidopsis thaliana* | 241 | + | 6 | GCCACT | cis-acting regulatory element related to meristem expression |
|  | GATA-motif | *Solanum tuberosum* | 999 | + | 9 | AAGGATAAGG | part of a light responsive element |
|  | Gap-box | *Arabidopsis thaliana* | 163 | + | 9.5 | CAAATGAA(A/G)A | part of a light responsive element |
|  | TATA-box | *Helianthus annuus* | 1219 | - | 6 | TATAAA | core promoter element around -30 of transcription start |
|  | TATC-box | *Oryza sativa* | 680 | + | 7 | TATCCCA | cis-acting element involved in gibberellin-responsiveness |
|  | TCT-motif | *Arabidopsis thaliana* | 1411 | + | 6 | TCTTAC | part of a light responsive element |
|  | MBS | *Arabidopsis thaliana* | 1044 | - | 6 | CAACTG | MYB binding site involved in drought-inducibility |
| ***PaCOL14*** | ABRE | *Arabidopsis thaliana* | 1336 | - | 6 | CACGTG | cis-acting element involved in the abscisic acid responsiveness |
|  | ARE | *Zea mays* | 1223 | + | 6 | AAACCA | cis-acting regulatory element essential for the anaerobic induction |
|  | Box 4 | *Petroselinum crispum* | 88 | + | 6 | ATTAAT | part of a conserved DNA module involved in light responsiveness |
|  | Box II | *Petroselinum crispum* | 1335 | + | 9 | CCACGTGGC | part of a light responsive element |
|  | CAAT-box | *Arabidopsis thaliana* | 277 | - | 5 | CCAAT | common cis-acting element in promoter and enhancer regions |
|  | CGTCA-motif | *Hordeum vulgare* | 1355 | + | 5 | CGTCA | cis-acting regulatory element involved in the MeJA-responsiveness |
|  | G-Box | *Pisum sativum* | 507 | - | 6 | CACGTT | cis-acting regulatory element involved in light responsiveness |
|  | G-box | *Lycopersicon esculentum* | 1335 | + | 10.5 | ACACGTG(G/t)CACC | cis-acting regulatory element involved in light responsiveness |
|  | GATA-motif | *Arabidopsis thaliana* | 1164 | - | 10 | AAGATAAGATT | part of a light responsive element |
|  | GC-motif | *Zea mays* | 1345 | + | 6 | CCCCCG | enhancer-like element involved in anoxic specific inducibility |
|  | HMG-TATA-region | *Lycopersicon esculentum* | 891 | - | 14 | CTATAAATGCATTTC | cis-acting regulatory element |
|  | I-box | *Zea mays* | 1188 | - | 9 | gGATAAGGTG | part of a light responsive element |
|  | MBS | *Arabidopsis thaliana* | 1114 | - | 6 | CAACTG | MYB binding site involved in drought-inducibility |
|  | TATA-box | *Oryza sativa* | 742 | + | 7 | TACAAAA | core promoter element around -30 of transcription start |
|  | TATC-box | *Oryza sativa* | 1193 | + | 7 | TATCCCA | cis-acting element involved in gibberellin-responsiveness |
|  | TCA-element | *Nicotiana tabacum* | 713 | + | 9 | CCATCTTTTT | cis-acting element involved in salicylic acid responsiveness |
|  | TGA-element | *Brassica oleracea* | 57 | - | 6 | AACGAC | auxin-responsive element |
|  | TGACG-motif | *Hordeum vulgare* | 1355 | - | 5 | TGACG | cis-acting regulatory element involved in the MeJA-responsiveness |
| ***PaCOL15*** | AE-box | *Arabidopsis thaliana* | 1330 | - | 8 | AGAAACAA | part of a module for light response |
|  | ARE | *Zea mays* | 570 | - | 6 | AAACCA | cis-acting regulatory element essential for the anaerobic induction |
|  | AT-rich element | *Glycine max* | 663 | - | 10 | ATAGAAATCAA | binding site of AT-rich DNA binding protein (ATBP-1) |
|  | ATCT-motif | *Pisum sativum* | 659 | - | 9 | AATCTAATCC | part of a conserved DNA module involved in light responsiveness |
|  | AuxRR-core | *Nicotiana tabacum* | 1458 | - | 7 | GGTCCAT | cis-acting regulatory element involved in auxin responsiveness |
|  | CAAT-box | *Arabidopsis thaliana* | 544 | - | 5 | CCAAT | common cis-acting element in promoter and enhancer regions |
|  | CAT-box | *Arabidopsis thaliana* | 519 | - | 6 | GCCACT | cis-acting regulatory element related to meristem expression |
|  | GATA-motif | *Solanum tuberosum* | 524 | - | 9 | AAGGATAAGG | part of a light responsive element |
|  | GCN4_motif | *Oryza sativa* | 1058 | - | 7 | TGAGTCA | cis-regulatory element involved in endosperm expression |
|  | GT1-motif | *Arabidopsis thaliana* | 1030 | + | 6 | GGTTAA | light responsive element |
|  | I-box | *Triticum aestivum* | 524 | - | 8 | AGATAAGG | part of a light responsive element |
|  | LTR | *Hordeum vulgare* | 749 | + | 6 | CCGAAA | cis-acting element involved in low-temperature responsiveness |
|  | MBS | *Arabidopsis thaliana* | 302 | + | 6 | CAACTG | MYB binding site involved in drought-inducibility |
|  | TATA-box | *Pisum sativum* | 274 | - | 7 | TATAAAA | core promoter element around -30 of transcription start |
|  | TCA-element | *Brassica oleracea* | 917 | - | 9 | TCAGAAGAGG | cis-acting element involved in salicylic acid responsiveness |
|  | TCT-motif | *Arabidopsis thaliana* | 225 | + | 6 | TCTTAC | part of a light responsive element |
|  | circadian | *Lycopersicon esculentum* | 1262 | + | 9 | CAAAGATATC | cis-acting regulatory element involved in circadian control |

**Table S3** Functional analysis of *PaCOL* genes on the basis of Gene Ontology (GO) terms assigned to various genes using BLAST2GO tool. GO terms enrichments in 3 different categories i.e. i) Cellular Component, ii) Molecular Function and iii) Biological Process were predicted

| **Genes Name** | **Go: Molecular Function** | **GO: Biological Process** | **Go: Cellular Component** |
| --- | --- | --- | --- |
| ***PaCOL1*** | Zinc ion binding, transition metal ion binding, metal ion binding, cation binding, ion binding (GO:0008270) | Circadian rhythm (Go:0007623) | Nucleous (GO: 0005730) |
| ***PaCOL2*** | Transcription regulatory region DNA binding, regulatory region DNA binding, regulatory region nucleic acid binding, DNA binding, nucleic acid binding, organic cyclic compound binding, heterocyclic compound binding (GO: 044212) | Regulation of transcription (GO:0006355) | Nucleous (GO: 0005634) |
| ***PaCOL3*** | Zinc ion binding, transition metal ion binding, metal ion binding, cation binding, ion binding (GO:0008270) | Response to temperature stimulus (GO: 0009266) | Nucleous (GO: 0005634) |
| ***PaCOL4*** | Zinc ion binding, transition metal ion binding, metal ion binding, cation binding, ion binding (GO:0008270) | Regulation of flower Development  (GO: 0009909) | Nucleous (GO: 0005730) |
| ***PaCOL5*** | Zinc ion binding, transition metal ion binding, metal ion binding, cation binding, ion binding (GO:0008270) | Response to salt stress (GO:0009651) | Nucleous (GO: 0005634) |
| ***PaCOL6*** | Zinc ion binding, transition metal ion binding, metal ion binding, cation binding, ion binding (GO:0008270) | Response to stress (GO: 0006950) | Nucleous (GO: 0005634) |
| ***PaCOL7*** | Zinc ion binding, transition metal ion binding, metal ion binding, cation binding, ion binding (GO:0008270 | Response to stress (GO:0006950) | Nucleous (GO: 0005634) |
| ***PaCOL8*** | Zinc ion binding, transition metal ion binding, metal ion binding, cation binding, ion binding (GO:0008270) | Regulation of flower development (GO: 0009909) | Nucleous (GO: 0005634 |
| ***PaCOL9*** | Zinc ion binding, transition metal ion binding, metal ion binding, cation binding, ion binding (GO:0008270) | Response to endogenous stimulus (GO: 0009719) | Nucleous (GO: 0005634 |
| ***PaCOL10*** | Zinc ion binding, transition metal ion binding, metal ion binding, cation binding, ion binding (GO:0008270) | Circadian rhythm (GO: 0007623) | Nucleous (GO: 0005634 |
| ***PaCOL11*** | Zinc ion binding, transition metal ion binding, metal ion binding, cation binding, ion binding (GO:0008270) | Regulation of photomorphogenesis (GO: 0010099) | Nucleous (GO: 0005634 |
| ***PaCOL12*** | Zinc ion binding, transition metal ion binding, metal ion binding, cation binding, ion binding (GO:0008270) | Response to salt stress (GO: 0009651) | Nucleous (GO: 0005634 |
| ***PaCOL13*** | Transcription regulatory region DNA binding, regulatory region DNA binding, regulatory region nucleic acid binding, DNA binding, nucleic acid binding, organic cyclic compound binding, heterocyclic compound binding (GO: 044212) | Regulation of flower development (GO: 0009909) | Nucleous (GO: 0005634 |
| ***PaCOL14*** | Zinc ion binding, transition metal ion binding, metal ion binding, cation binding, ion binding (GO:0008270) | Response to temperature stimulus (GO: 0009266) | Nucleous (GO: 0005730 |
| ***PaCOL15*** | Zinc ion binding, transition metal ion binding, metal ion binding, cation binding, ion binding (GO:0008270) | Photomorphogenesis (GO:0009640) | Nucleous (GO: 0005634 |

**Table S4** Ks, Ka, and Ka/Ks ratios calculated for paralogous pairs of petunia *COL* genes.

| **Paralogous Pair** | **Ks** | **Ka** | **ka/ks** | **Selection Pressure** | **Time (Mya)** |
| --- | --- | --- | --- | --- | --- |
| *PaCOL1-PaCOL2* | 0.6130 | 0.3088 | 0.5038 | Purifying selection | 20.4 |
| *PaCOL6-PaCOL9* | 0.7517 | 0.1972 | 0.2623 | Purifying selection | 25.23 |
| *PaCOL13-PaCOL6* | 0.6382 | 0.2020 | 0.3165 | Purifying selection | 21.27 |
| *PaCOL13-PaCOL9* | 0.6061 | 0.1758 | 0.2900 | Purifying selection | 20.20 |
| *PaCOL5-PaCOL12* | 1.0429 | 0.1787 | 0.1713 | Purifying selection | 34.76 |
| *PaCOL10-PaCOL15* | 1.3146 | 0.2984 | 0.2270 | Purifying selection | 43.82 |

**Table S5** Sequence analysis of petunia COL proteins identified in *Petunia infrata* genome

| **Gene name** | **Locus ID** | **ORF (bp)** | **Proteins** | | | | |  |
| --- | --- | --- | --- | --- | --- | --- | --- | --- |
|  |  |  | **Length (aa)** | **Domain Start-end (aa)** | | **MW(kDa)** | **p^I^** | **GRAVY** |
|  |  |  |  | **BBOX** | **CCT** |  |  |  |
| *PiCOL1* | [Peinf101Ctg13801653g00004](http://planttfdb.gao-lab.org/tf.php?sp=Pin&did=Peinf101Ctg13801653g00004.1) | 1302 | 434 | 4-47,47-72 | 392-422 | 48.15 | 5.87 | -0.540 |
| *PiCOL2* | [Peinf101Scf00023g02011](http://planttfdb.gao-lab.org/tf.php?sp=Pin&did=Peinf101Scf00023g02011.1) | 1224 | 407 | 18-61 | 353-395 | 46.81 | 5.51 | -0.843 |
| *PiCOL3* | [Peinf101Scf00039g06007](http://planttfdb.gao-lab.org/tf.php?sp=Pin&did=Peinf101Scf00039g06007.1) | 1389 | 462 | 4-47,47-73 | 406-449 | 51.04 | 5.39 | -0.477 |
| *PiCOL4* | [Peinf101Scf00055g00010](http://planttfdb.gao-lab.org/tf.php?sp=Pin&did=Peinf101Scf00055g00010.1) | 1239 | 412 | 5-47,47-90 | 355-398 | 45.21 | 5.16 | -0.568 |
| *PiCOL5* | [Peinf101Scf00131g09008](http://planttfdb.gao-lab.org/tf.php?sp=Pin&did=Peinf101Scf00131g09008.1) | 1242 | 413 | 4-47,47-90 | 359-399 | 45.45 | 6.04 | -0.630 |
| *PiCOL6* | [Peinf101Scf00264g05032](http://planttfdb.gao-lab.org/tf.php?sp=Pin&did=Peinf101Scf00264g05032.1) | 1269 | 422 | 4-47 | 366-409 | 45.62 | 5.48 | -0.402 |
| *PiCOL7* | [Peinf101Scf00276g05013](http://planttfdb.gao-lab.org/tf.php?sp=Pin&did=Peinf101Scf00276g05013.1) | 1074 | 357 | 12-55,55-98 | 294-336 | 39.50 | 5.54 | -0.509 |
| *PiCOL8* | [Peinf101Scf00359g05021](http://planttfdb.gao-lab.org/tf.php?sp=Pin&did=Peinf101Scf00359g05021.1) | 957 | 318 | 13-56,56-99 | 249-291 | 35.60 | 5.37 | -0.685 |
| *PiCOL9* | [Peinf101Scf00889g02024](http://planttfdb.gao-lab.org/tf.php?sp=Pin&did=Peinf101Scf00889g02024.1) | 1395 | 464 | 13-56,56-99 | 416-459 | 51.27 | 6.85 | -0.717 |
| *PiCOL10* | [Peinf101Scf01086g01009](http://planttfdb.gao-lab.org/tf.php?sp=Pin&did=Peinf101Scf01086g01009.1) | 1302 | 434 | 4-47,47-72 | 392-422 | 48.15 | 5.87 | -0.540 |
| *PiCOL11* | [Peinf101Scf01103g05015](http://planttfdb.gao-lab.org/tf.php?sp=Pin&did=Peinf101Scf01103g05015.1) | 1122 | 373 | 19-62,62-103 | 322-365 | 41.99 | 5.50 | -0.766 |
| *PiCOL12* | [Peinf101Scf01775g01026](http://planttfdb.gao-lab.org/tf.php?sp=Pin&did=Peinf101Scf01775g01026.1) | 1242 | 413 | 4-47,47-90 | 356-399 | 45.35 | 5.53 | -0.592 |
| *PiCOL13* | [Peinf101Scf01850g00016](http://planttfdb.gao-lab.org/tf.php?sp=Pin&did=Peinf101Scf01850g00016.1) | 1011 | 336 | 63-105,23-62 | 262-304 | 36.66 | 6.45 | -0.271 |
| *PiCOL14* | [Peinf101Scf02263g03015](http://planttfdb.gao-lab.org/tf.php?sp=Pin&did=Peinf101Scf02263g03015.1) | 1314 | 437 | 18-61 | 388-430 | 49.36 | 5.41 | -0.729 |
| *PiCOL15* | [Peinf101Scf03311g00022](http://planttfdb.gao-lab.org/tf.php?sp=Pin&did=Peinf101Scf03311g00022.1) | 945 | 314 | 27-70,2-27 | 251-294 | 35.28 | 5.60 | -0.596 |
| *PiCOL16* | [Peinf101Scf03422g00031](http://planttfdb.gao-lab.org/tf.php?sp=Pin&did=Peinf101Scf03422g00031.1) | 1146 | 381 | 1-35 | 324-366 | 43.84 | 4.83 | -0.832 |
| *PiCOL17* | [Peinf101Scf04017g01047](http://planttfdb.gao-lab.org/tf.php?sp=Pin&did=Peinf101Scf04017g01047.1) | 1224 | 407 | 18-61 | 350-392 | 46.41 | 4.97 | -0.777 |
| *PiCOL18* | [Peinf101Scf12610g00001](http://planttfdb.gao-lab.org/tf.php?sp=Pin&did=Peinf101Scf12610g00001.1) | 915 | 304 | 5-47,47-90 | 241-284 | 34.40 | 6.09 | -0.564 |

**Table S6** Parameter and program versions used in bioinformatics analysis

| **Sl no.** | **Program Name** | **Link** | **Version** | **Parameters** |
| --- | --- | --- | --- | --- |
| 1 | ORF finder tool | <https://www.ncbi.nlm.nih.gov/orffinder/> | - | Minimal ORF 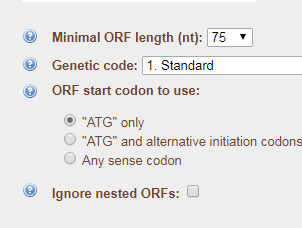 |
| 2 | Expasy-Protopram | <https://web.expasy.org/protparam/> | - | <https://web.expasy.org/protparam/protparam-doc.html> |
| 3 | Gene Structure Display Server (GSDS) | <http://gsds.cbi.pku.edu.cn/> | V2.0 | 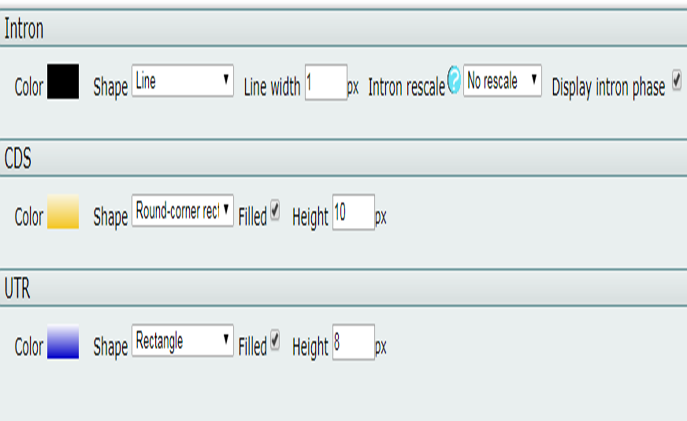 |
| 4 | Genedoc | <http://www.nrbsc.org/gfx/genedoc/ebinet.htm> | v2.7 | - |
| 5 | Multiple EM for Motif Elicitation (MEME) | <http://meme-suite.org/> | V5.3 | 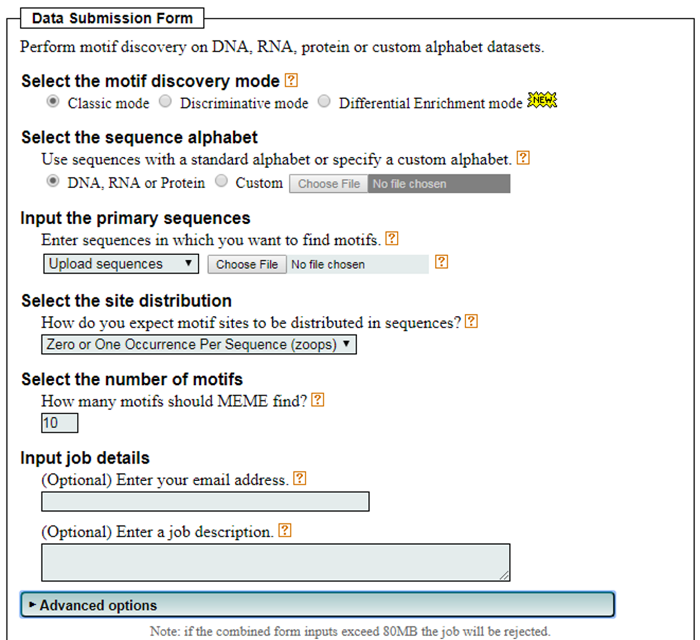 |
| 6 | PlantCARE | <http://bioinformatics.psb.ugent.be/webtools/plantcare/html/> | - | - |
| 7 | Blast2GO | <https://www.blast2go.com> | - | - |
| 8 | NCBI | <https://www.ncbi.nlm.nih.gov/> | - | - |
| 9 | Sol Genomics databases | <https://www.sgn.cornell.edu/> | - | - |
| 10 | Phytozome database | <https://phytozome.jgi.doe.gov/pz/portal.html> | V12.1 |  |
| 11 | InterProScan | <http://www.ebi.ac.uk/Tools/pfa/iprscan5/> | - | - |
| 12 | SMART | <http://smart.embl-heidelberg.de/> | - | - |
| 13 | TAIR | <http://www.arabidopsis.org/> | - | - |
| 14 | TIGR | <http://rice.plantbiology.msu.edu/> | - | - |
| 15 | Nei and Gojobori | <http://cbb.big.ac.cn/software> | v2.0.3 | - |
| 16 | OrthoMCL | <https://orthomcl.org/orthomcl/app> | v2.0.3 | 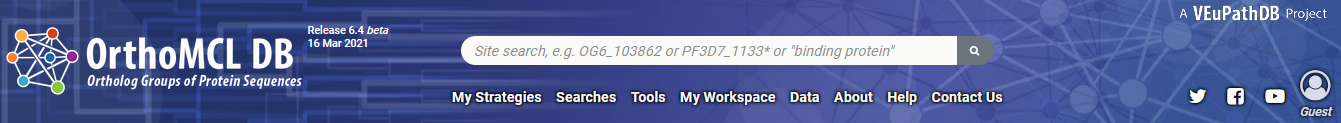 - |
| 17 | MINITAB |  | v18 | - One way ANNOVA - Tukey comparison procedures assuming equal variance - 95% confidence level - Two sided confidence level - Error rate of comparison 5 |
| 18 | MEGA | 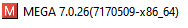 | v7.0 | 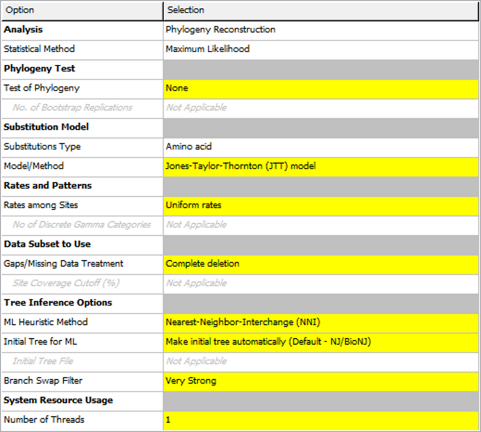 |
