# Supplementary material for: Genome-wide identification, genomic organization, and expression profiling of the CONSTANS-like (COL) gene family in petunia under multiple stresses
Source: BMC Genomics. 2021 Oct 8;22:727. doi: 10.1186/s12864-021-08019-w (PMC8499527; doi:10.1186/s12864-021-08019-w)
Supplement: Supplementary file 3 — Additional file 3: Figure S1. Sequence alignment of COL proteins from petunia, Arabidopsis and rice according to the presence of domains. Each letter represents one amino acid, and the left column corresponds to the name of the gene. The BBOX1 and BBOX2 domains are indicated by the blue and green line and CCT domains are indicated by the red line respectively. The red region indicates residues conserved only in the BBOX1 domain, the green region indicates residues conserved in the BBOX2 domain and the indigo region indicates residues conserved in the CCT domain of CO-like proteins. Figure S2. Domain architecture of PaCOL proteins. BBOX and CCT domains are indicated by mint and purple color respectively. The black middle region represents the diverge region of COL proteins. Figure S3. Analysis of motif composition in PaCOL proteins. Different motifs were represented by different color boxes. MEME database was used for motif analysis with the complete amino acid sequences of PaCOL proteins. Figure S4. Phylogenetic analysis of the COL proteins of Petunia inflata, Arabidosis, rice, maize, tomato and Physcomitrella patens. The phylogenetic tree was established with entire protein sequences from the above plant species by MEGA 7.0 software using the Maximum likelihood method following the complete deletion procedure. The numbers on the branches indicate bootstrap support values from 1000 replications. The scale represents the units of the number of amino acid substitutions per site. Figure S5. Analysis of gene structure in PiCOL. Blue boxes, black boxes, and red lines indicate exons, upstream/downstream, and introns, respectively. Figure S6. Electrophoresis gel image of different organ samples. [file 12864_2021_8019_MOESM3_ESM.pptx]

## Slide 1
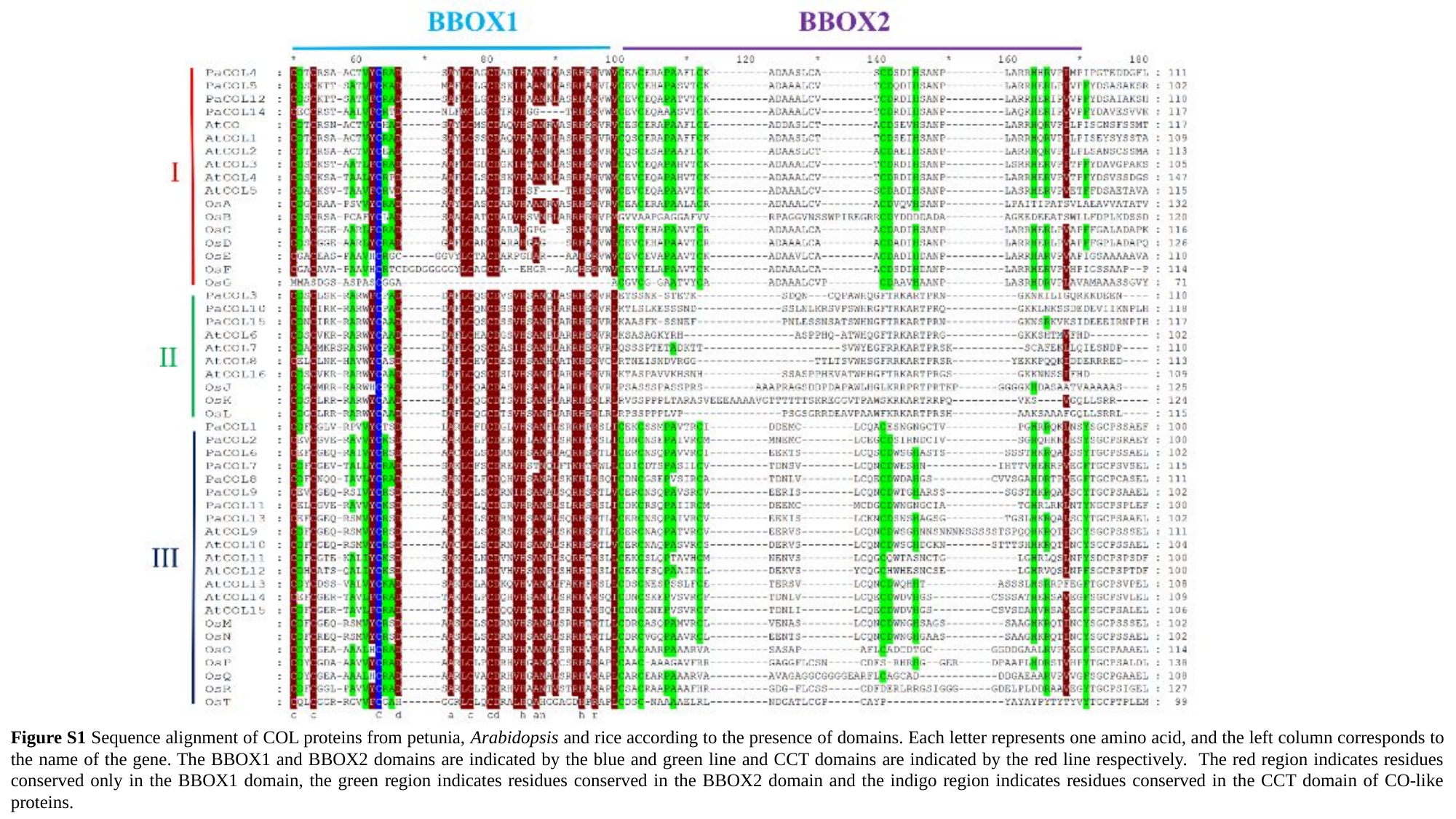

Figure S1 Sequence alignment of COL proteins from petunia, Arabidopsis and rice according to the presence of domains. Each letter represents one amino acid, and the left column corresponds to the name of the gene. The BBOX1 and BBOX2 domains are indicated by the blue and green line and CCT domains are indicated by the red line respectively. The red region indicates residues conserved only in the BBOX1 domain, the green region indicates residues conserved in the BBOX2 domain and the indigo region indicates residues conserved in the CCT domain of CO-like proteins.

## Slide 2
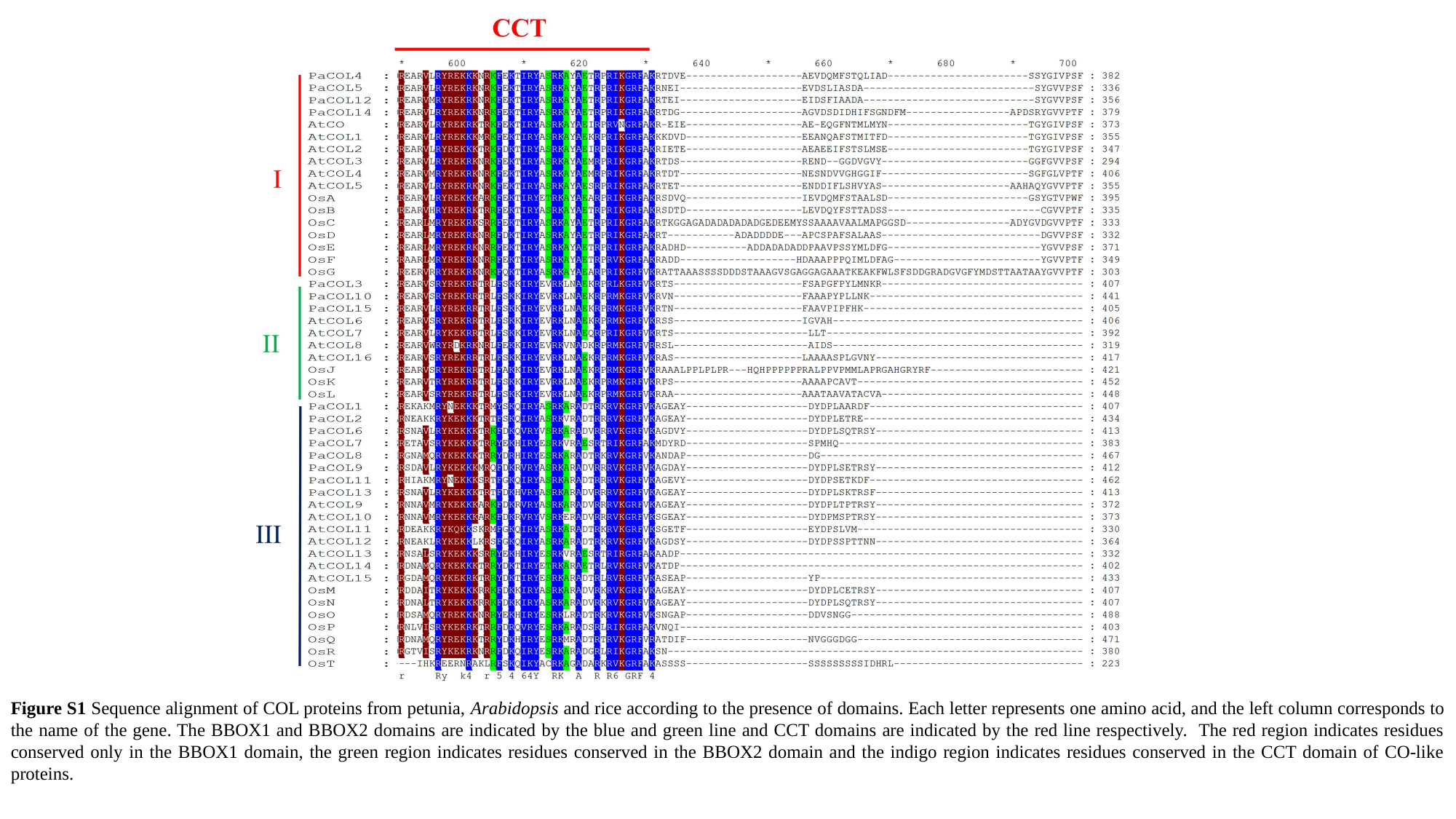

Figure S1 Sequence alignment of COL proteins from petunia, Arabidopsis and rice according to the presence of domains. Each letter represents one amino acid, and the left column corresponds to the name of the gene. The BBOX1 and BBOX2 domains are indicated by the blue and green line and CCT domains are indicated by the red line respectively. The red region indicates residues conserved only in the BBOX1 domain, the green region indicates residues conserved in the BBOX2 domain and the indigo region indicates residues conserved in the CCT domain of CO-like proteins.

## Slide 3
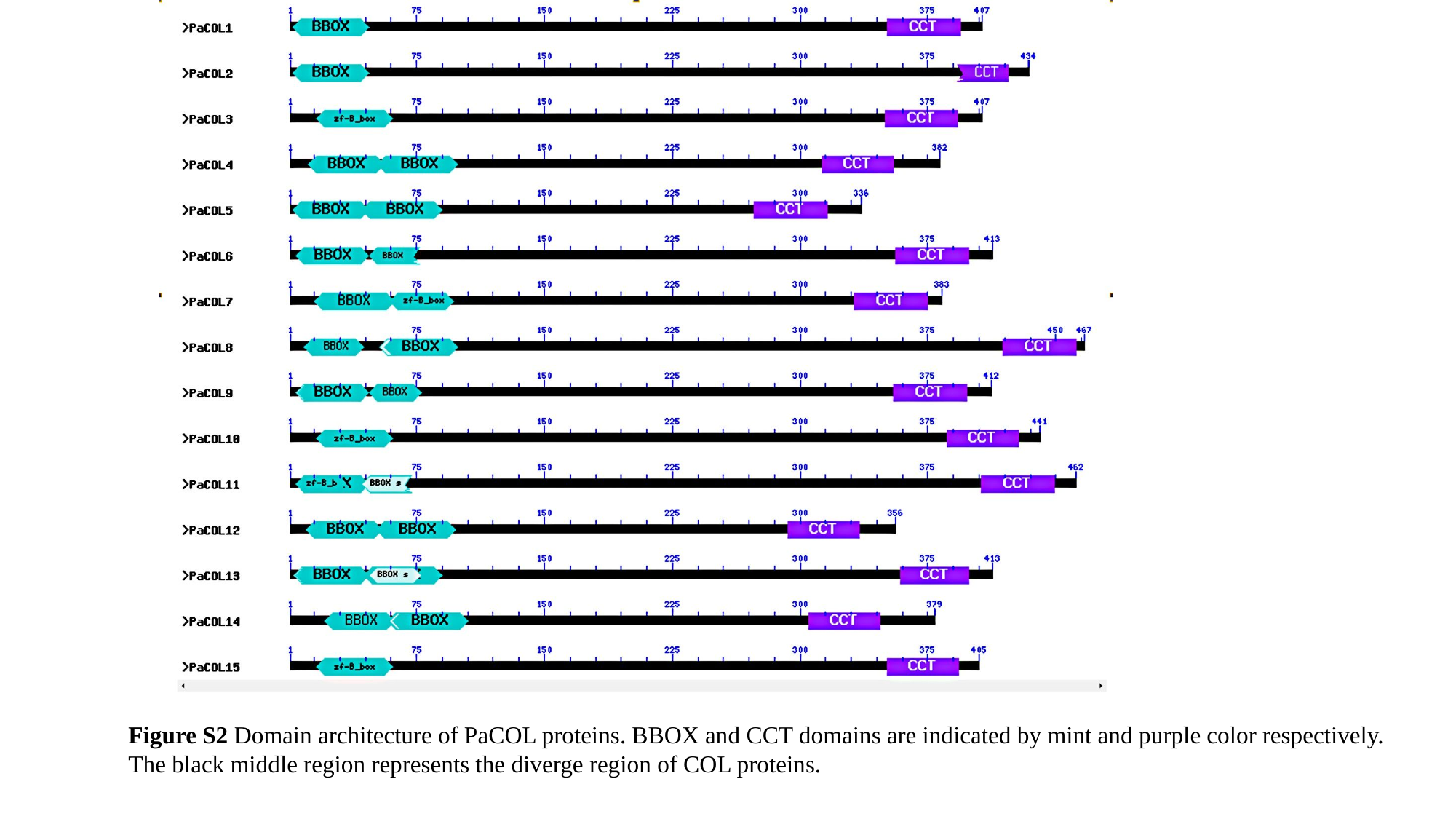

Figure S2 Domain architecture of PaCOL proteins. BBOX and CCT domains are indicated by mint and purple color respectively. The black middle region represents the diverge region of COL proteins.

## Slide 4
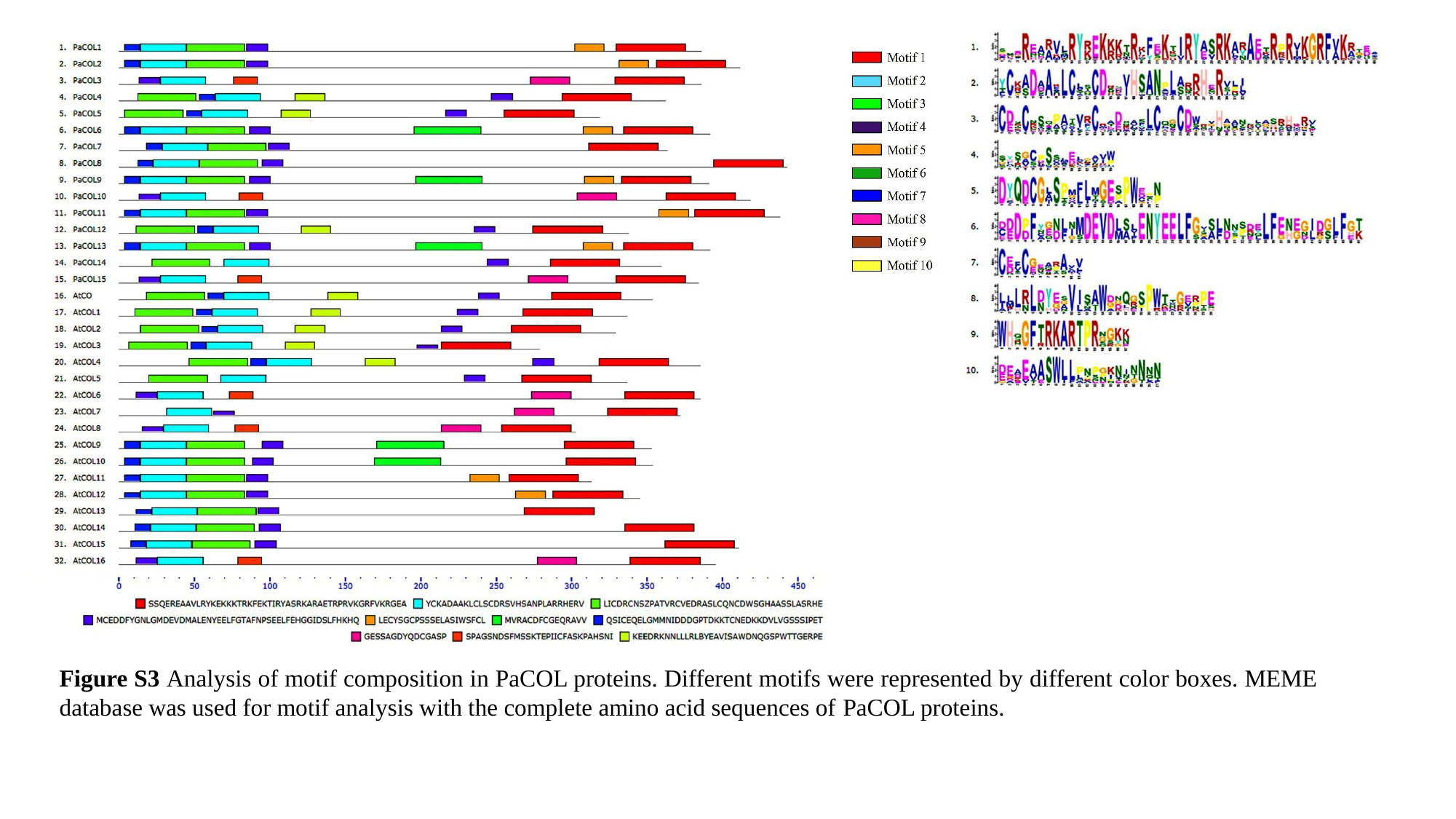

Figure S3 Analysis of motif composition in PaCOL proteins. Different motifs were represented by different color boxes. MEME database was used for motif analysis with the complete amino acid sequences of PaCOL proteins.

## Slide 5
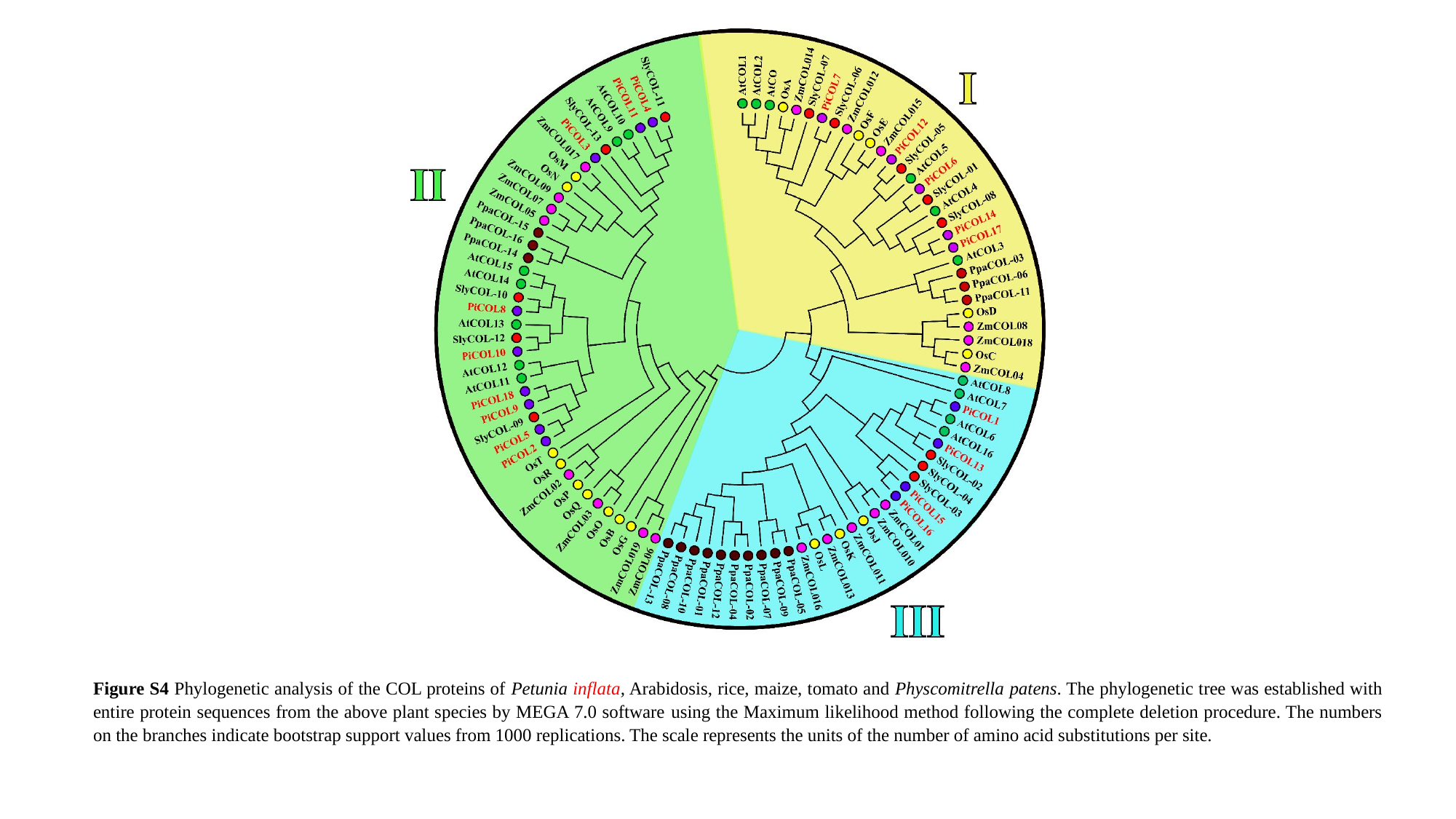

Figure S4 Phylogenetic analysis of the COL proteins of Petunia inflata, Arabidosis, rice, maize, tomato and Physcomitrella patens. The phylogenetic tree was established with entire protein sequences from the above plant species by MEGA 7.0 software using the Maximum likelihood method following the complete deletion procedure. The numbers on the branches indicate bootstrap support values from 1000 replications. The scale represents the units of the number of amino acid substitutions per site.

## Slide 6
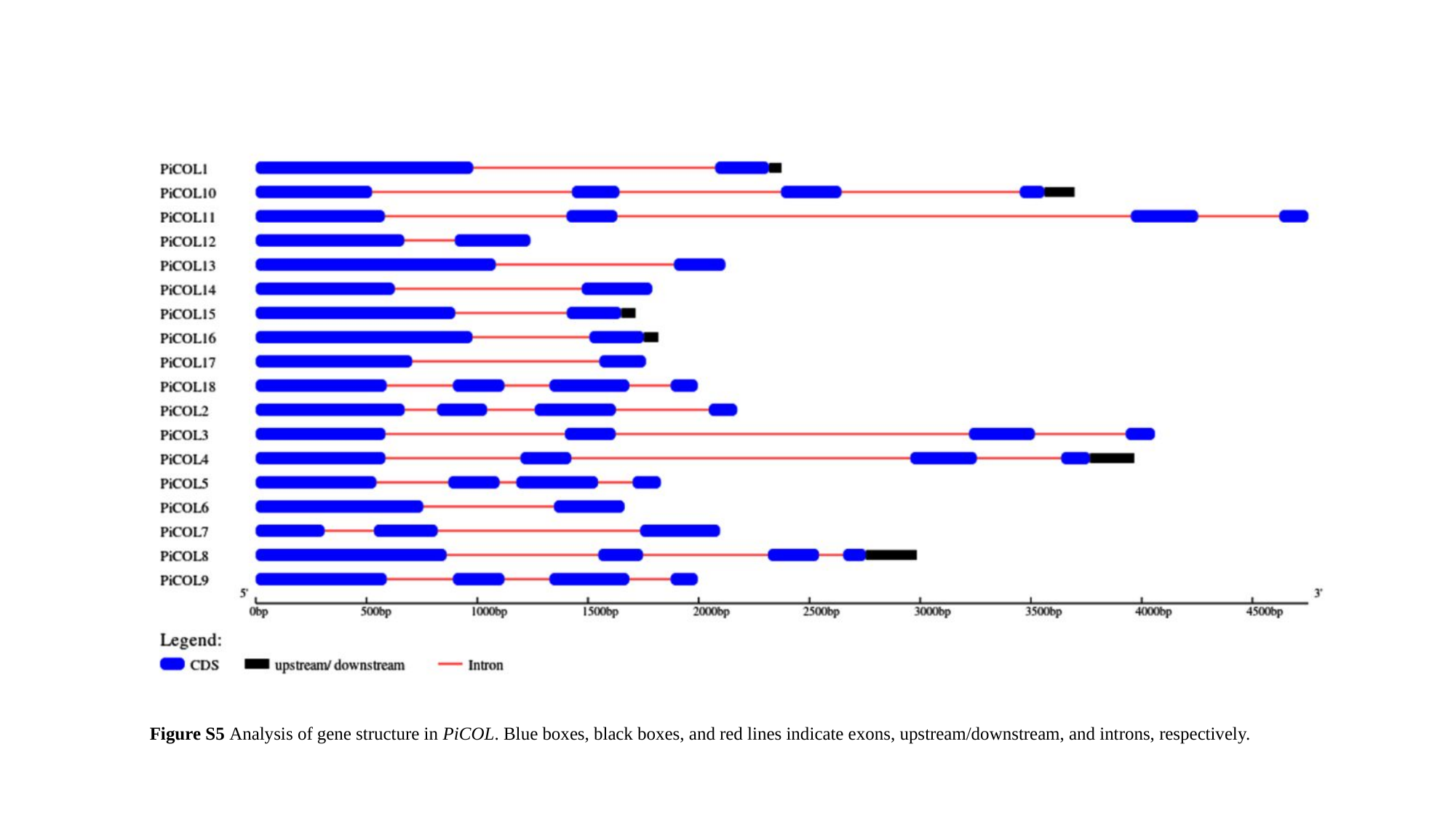

Figure S5 Analysis of gene structure in PiCOL. Blue boxes, black boxes, and red lines indicate exons, upstream/downstream, and introns, respectively.

## Slide 7
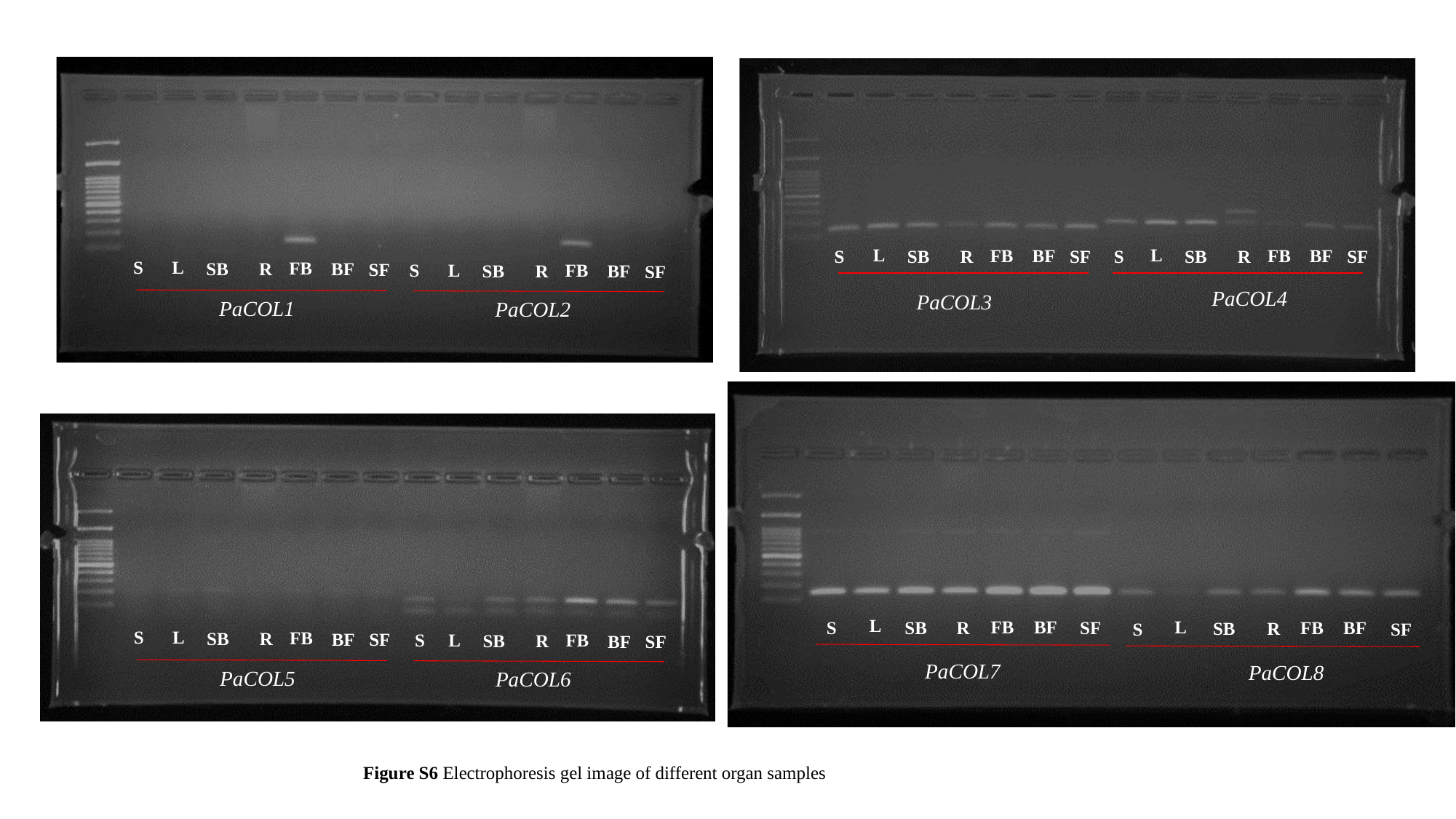

S
L
FB
SB
R
BF
SF
FB
S
L
SB
R
BF
SF
PaCOL1
PaCOL2
L
BF
FB
SB
R
SF
S
L
BF
FB
SB
R
SF
S
PaCOL4
PaCOL3
L
BF
FB
SB
R
SF
S
L
BF
FB
SB
R
SF
S
PaCOL7
PaCOL8
S
L
FB
SB
R
BF
SF
FB
S
L
SB
R
BF
SF
PaCOL5
PaCOL6
Figure S6 Electrophoresis gel image of different organ samples

## Slide 8
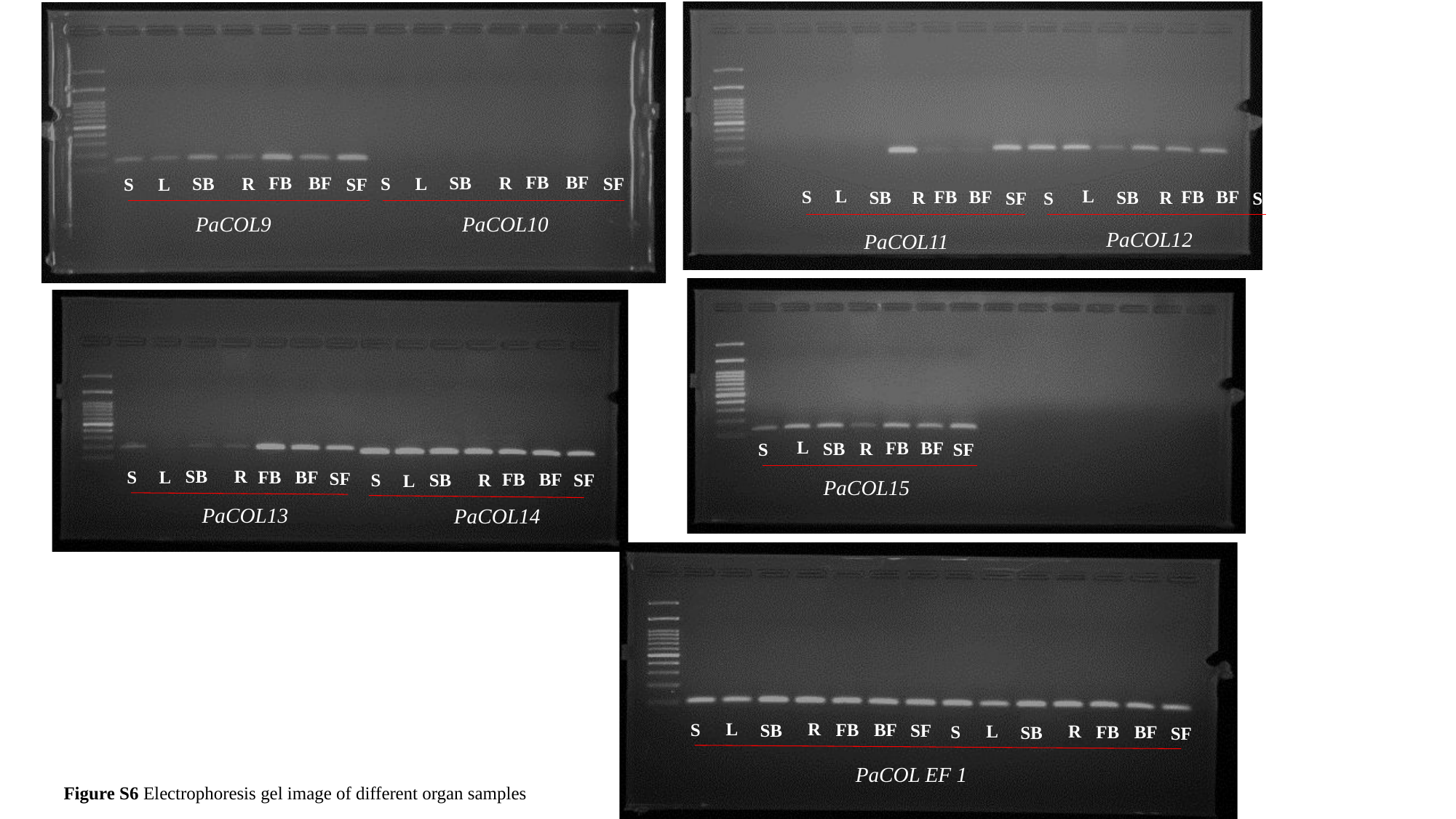

L
BF
FB
S
SB
R
SF
L
BF
FB
SB
R
SF
S
PaCOL12
PaCOL11
BF
FB
SB
R
SF
S
L
BF
FB
SB
R
SF
S
L
PaCOL9
PaCOL10
L
BF
FB
SB
R
SF
S
PaCOL15
SB
R
BF
FB
S
L
SF
BF
FB
SB
R
SF
S
L
PaCOL13
PaCOL14
L
R
BF
FB
S
SB
SF
L
R
BF
FB
S
SB
SF
PaCOL EF 1
Figure S6 Electrophoresis gel image of different organ samples
